# AMPKα2 controls the anti-atherosclerotic effects of fish oils by modulating the SUMOylation of GPR120

**DOI:** 10.1038/s41467-022-34996-x

**Published:** 2022-12-13

**Authors:** Cheng-hui Yan, Hai-Wei Liu, Xiao-xiang Tian, Jiayin Li, Ye Ding, Yi Li, Zhu Mei, Ming-Hui Zou, Ya-ling Han

**Affiliations:** 1 Cardiovascular Research Institute and Department of Cardiology, General Hospital of Northern Theater Command, Shenyang, 110016 China; 2 Center for Molecular and Translational Medicine, Georgia State University, Atlanta, GA 30303 USA

**Keywords:** Cardiology, Cardiovascular diseases

## Abstract

Consuming fish oils (FO) is linked to reduced risk of cardiovascular disease in certain populations. However, FO failed to exhibit therapeutic effects in some patients with cardiovascular disease. This study aimed to determine the possible reasons for the inconsistent effects of FO. AMP-activated protein kinase (AMPK) α2 is an important energy metabolic sensor, which was reported to involve in FO mediated regulation of lipid and glucose metabolism. In an in vivo study, FO administration significantly reduced the aortic lesions and inflammation in the *Ldlr*^−/−^ mouse model of atherosclerosis, but not in *Ldlr*^−/−^/*Prkaa2*^−/−^and *Ldlr*^−/−^/*Prkaa2*^−/−Sm22Cre^ mice. Mechanistically, inactivation of AMPKα2 increased the SUMOylation of the fatty acid receptor GPR120 to block FO-induced internalization and binding to β-arrestin. In contrast, activation of AMPKα2 can phosphorylate the C-MYC at Serine 67 to inhibit its trans-localization into the nuclei and transcription of SUMO-conjugating E2 enzyme UBC9 and SUMO2/3 in vascular smooth muscle cells (VSMCs), which result in GPR120 SUMOylation. In human arteries, AMPKα2 levels were inversely correlated with UBC9 expression. In a cohort of patients with atherosclerosis, FO concentrations did not correlate with atherosclerotic severity, however, in a subgroup analysis a negative correlation between FO concentrations and atherosclerotic severity was found in patients with higher AMPKα2 levels. These data indicate that AMPKα2 is required for the anti-inflammatory and anti-atherosclerotic effects of FO.

## Abstract (web summary)

Consuming fish oil is linked to reduced risk of cardiovascular disease in some populations, but the effects are not consistent across studies. Here the authors report that smooth muscle cell AMPKα2 is required for the protective effects of fish oil on atherosclerosis in mice, and acts via post-translational regulation of the fatty acid receptor GPR120.

## Introduction

Cardiovascular disease (CVD) is a leading cause of morbidity and mortality worldwide^1^. One of the most effective approaches for preventing CVDs has been dietary intervention^2^. The consumption of fish oils (FO) containing omega-3 long-chain polyunsaturated fatty acids such as docosahexaenoic acid (DHA; C22:6n3) and eicosapentaenoic acid (EPA; C20:5n3) is linked to a reduction of CVDs, including coronary artery disease (CAD)^3,4^. Several early epidemiological studies including a Greenland Inuit population and Okinawa islanders^5,6^ showed that an abundance of omega-3 fatty acids in diets is inversely associated with CAD mortality. Both randomized clinical trials and animal studies has demonstrated that FO has numerous CVD protective effects, including lowering triglycerides^7^, improving endothelial function^8^, reducing arterial wall stiffness^9^, and suppressing inflammation^10^. However, several prospective clinical studies of FO have reported ineffective data on CAD in recent years^11–15^. This inconsistent finding implicates that the beneficial effect mechanism of FO on CAD treatment has not been elucidated.

Accumulating evidence suggests that omega-3 fatty acids have anti-inflammatory and insulin-sensitizing effects via the G protein-coupled receptor 120 (GPR120), also called free fatty acid receptor 4, which belongs to the rhodopsin subfamily of G protein-coupled receptors^16^. Consistent with the pleiotropic effects of FO, GPR120 improves many aspects of metabolic homeostasis, such as insulin sensitivity, macrophage function, and the suppression of inflammasome activation, whereas its disruption in both mice and humans is linked to the anti-inflammatory effects of FO under conditions of metabolic dysfunction^17^. GPR120 agonists trigger downstream signaling pathways for calcium mobilization, adenylyl cyclase inhibition, receptor phosphorylation, and receptor internalization^18,19^. Similar to other GPCRs, SUMOylation of cellular proteins is a prominent post-translational modification that regulates protein function, subcellular localization, and expression^20^. SUMO1, 2, and 3 reportedly regulate protein function, subcellular localization, and/or expression^21^. Whether GPR120 SUMOylation impacts FO anti-CVD remains largely unknown.

Many studies^20–23^ suggested that DHA and EPA, exhibit the important function in the regulation of glucose and lipid metabolism by partly activating the AMP-activated protein kinase (AMPK) function in vitro and in vivo. Our previous study showed that *Prkaa2* knock out (AMPKα2^−/−^) are more prone to atherosclerosis (AS) than *Prkaa1* knock out (AMPKα1^−/−^) mice model^24,25^. However, whether the inactivation of AMPK can block the FO beneficial effects on CAD patients still unclear.

In the work, we investigate that inactivation of AMPKα2 blocks the anti-atherosclerotic effects of FO both in CAD patients and in mice model. Furthermore, suppression of AMPKα2 leads to the dysfunctions of GPR120 through increase of its SUMOylated modification.

## Results

### AMPKα2 is required for the anti-atherosclerotic effects of FO in mice

**Fig. 1:**
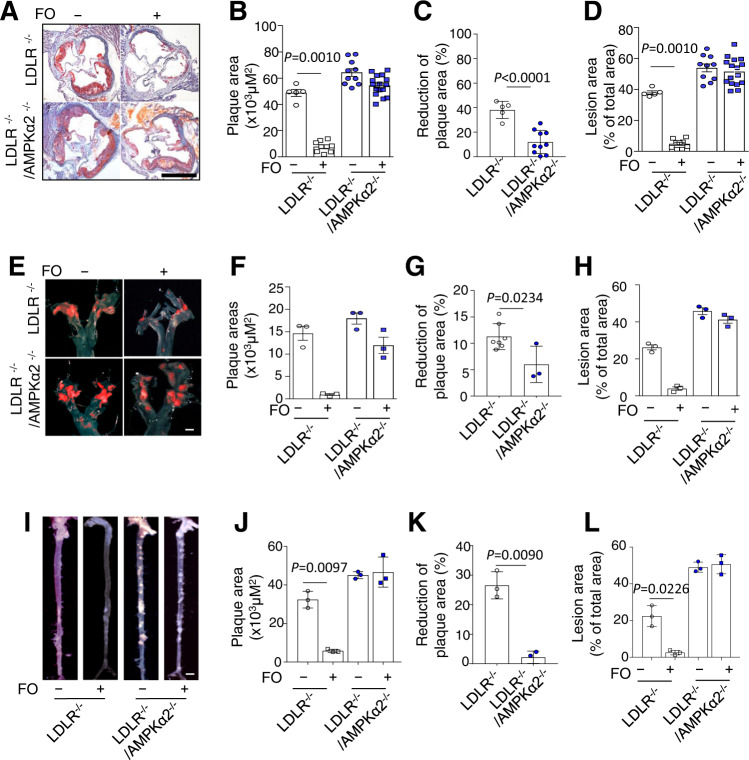
FO administration inhibited atherosclerotic plaque formation in LDLR^−/−^, but not LDLR^−/−^/AMPKα2^−/−^ mice. LDLR^−/−^/AMPKα2^−/−^ mice and their LDLR^−/−^ littermates were fed a western diet (containing 0.21% cholesterol) for 12 weeks with or without 5% FO administration. **A**, **E**, **I** Representative images of Oil Red O staining from each of four groups: LDLR^−/−^ (*n* = 11), LDLR^−/−^ + FO (*n* = 17), LDLR^−/−^/AMPKα2^−/−^ (*n* = 16), and LDLR^−/−^/AMPKα2^−/−^ + FO (*n* = 22). Scale bar, 200 μm. Quantification of aortic-lesion areas using Image-Pro plus software (**B**, **F**, **J**) and reduced plaque areas (**C**, **G**, **K**) between mice with and without FO treatment. **D**, **H**, **L** Percentages of lesion areas (ratio to lumen areas) of aortic roots were calculated for the four groups. *p* value vs LDLR^−/−^ by a two-sided Student’s *t* test. Data are presented as the mean ± s.e.m. WD western diet.

Consistent with the previous report^24^, the plaque areas in the aortic roots of LDLR^−/−^/AMPKα2^−/−^ mice were significantly larger than those of LDLR^−/−^ mice (Fig. 1A, B). A diet containing FO significantly reduced lesions in the aortic roots of LDLR^−/−^ mice to 81.2%, but this effect was attenuated in LDLR^−/−^/AMPKα2^−/−^ mice only a 10.3% reduction (Fig. 1C). FO significantly reduced the ratio of aortic plaques to the lumen in aortic roots in LDLR^−/−^/AMPKα2^−/−^ mice versus LDLR^−/−^ mice (Fig. 1D). Similar results were observed when the effects of FO in the aortic arches (Fig. 1E–H) and total aortic tissues (Fig. 1I–K) of LDLR^−/−^ and LDLR^−/−^/AMPKα2^−/−^ mice were evaluated. These results imply that the anti-atherosclerotic effect of FO requires AMPKα2 in vivo.

### AMPKα2 controls the anti-inflammatory but not the lipid-lowering effect of FO

**Fig. 2:**
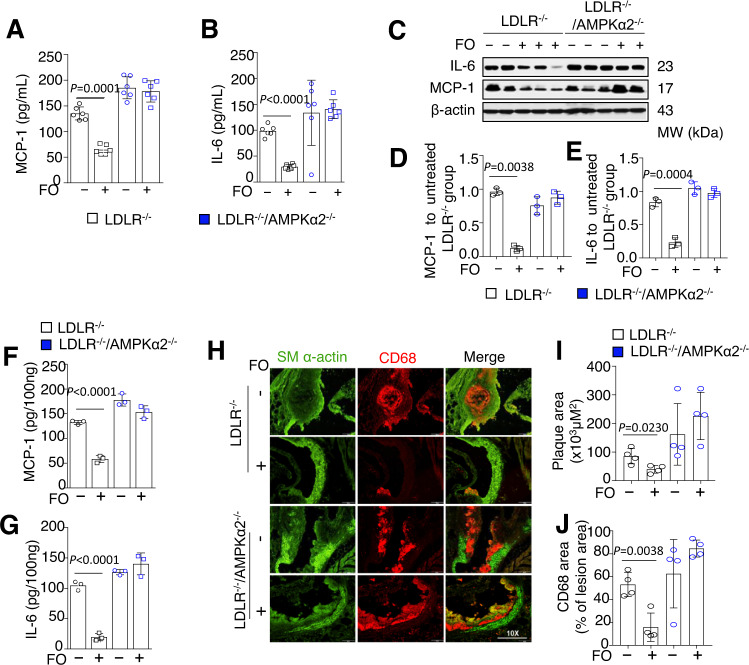
AMPKα2 was required for the anti-inflammatory effects of FO in vivo. Serum MCP-1 (**A**) and IL-6 (**B**) levels were measured in LDLR^−/−^ and LDLR^−/−^/AMPKα2^−/−^ mice fed a western diet with or without 5% FO treatment (*n* = 6). Representative western blot (**C**) and quantification of MCP-1 (**D**) and IL-6 (**E**) and protein levels in aortic tissues from LDLR^−/−^ and LDLR^−/−^/AMPKα2^−/−^ mice fed a western diet for 12 weeks, with or without 5% FO administration (*n* = 3), distinctly from loading controls. ELISAs for MCP-1 (**F**) and IL-6 (**G**) in aortic tissue lysates from LDLR^−/−^ and LDLR^−/−^/AMPKα2^−/−^ mice with or without 5% FO administration (*n* = 3). **H** Representative immunofluorescence images for SM α-actin and CD68 in aortic tissues of LDLR^−/−^ and LDLR^−/−^/AMPKα2^−/−^ mice, with or without 5% FO administration. **I**, **J** Quantification of CD68 area and ratio in aortic tissues of LDLR^−/−^ and LDLR^−/−^/AMPKα2^−/−^ mice, with or without 5% FO administration (*n* = 4). Scale bars, 200 μm. The data shown represent one of two separate experiments. All values are expressed as means ± s.e.m. *p* value vs LDLR^−/−^ mice by two-sided Student’s *t* tests. FO fish oils, MCP-1 monocyte chemoattractant protein-1, IL-6 interlukin-6.

FO administration did not alter body weight or serum glucose levels (Fig. S2A, B) but significantly lowered the levels of serum cholesterol and triglycerides (Fig. S2C, D) in both LDLR^−/−^ and LDLR^−/−^/AMPKα2^−/−^ mice. FO significantly lowered both macrophage chemoattractant protein-1 (MCP-1) and interlukin-6 (IL-6) levels in sera from LDLR^−/−^ but not from LDLR^−/−^/AMPKα2^−/−^ mice (Fig. 2A, B). Similarly, FO treatment dramatically inhibited the expression of IL-6 and MCP-1 in the aortic tissues of LDLR^−/−^ but not of LDLR^−/−^/AMPKα2^−/−^ mice (Fig. 2C–G). FO markedly lowered the levels of macrophage cell marker CD68 in aortic tissue of LDLR^−/−^ but not of LDLR^−/−^/AMPKα2^−/−^ mice (Fig. 2H–J), indicating that the anti-inflammatory effect of FO is AMPKα2-dependent.

### AMPKα2 specific deficiency in VSMCs ablates the FO anti-atherosclerotic effect in vivo

AMPKα2 is the isoform mainly expressed in the VSMCs of blood vessels^24^. Further, we used the LDLR^−/−^/AMPKα2^Sm22Cre^ mice to investigate the anti-atherosclerotic effect of FO. FO administration did not reduce plaque formation (Fig. S3A–C) or secretion of MCP-1 and IL-6 in sera (Fig. S3D, E), although it dramatically decreased serum TG and CHO levels (Fig. S3F, G) in LDLR^−/−^/AMPKα2^Sm22Cre^ mice fed a western diet for 12 weeks. It suggested that AMPKα2 is involved into the regulation of FO anti-inflammation in VSMCs.

### AMPKα2 regulates the anti-inflammatory effect of FO in primary cultured VSMCs

**Fig. 3:**
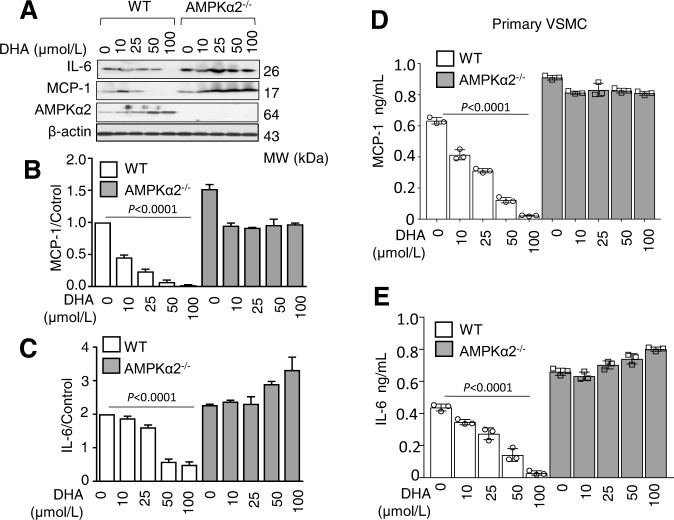
AMPKα2 was required for the anti-inflammatory effects of DHA in cultured VSMCs. **A** Representative western blot (**A**) and quantification for the expression of MCP-1 (**B**) and IL-6 (**C**) in PA (300 μM)-treated primary cultured WT and AMPKα2^−/−^ VSMCs with DHA (0, 10, 25, 50, 100 μM). The data shown represent one of at least two separate experiments (*n* = 2), distinctly from loading controls. ELISAs for MCP-1 (**D**) and IL-6 (**E**) in culture supernatants from WT or AMPKα2 deficient-VSMCs treated with or without PA (300 μM) and DHA (0, 10, 25, 50, 100 μM). The data shown represent one of three separate experiments (*n* = 3). DHA treatment group analysis by one-way ANOVA test. VSMC vascular smooth muscle cell, WT wild type, DHA docosahexaenoic acid, MCP-1 monocyte chemoattractant protein-1, IL-6 interlukin-6.

DHA treatment significantly lowered the palmitic acid (PA)-induced increases of IL-6 and MCP-1 in VSMCs from WT mice in a dose-dependent manner. In contrast, DHA did not suppress the PA-induced elevation of both IL-6 and MCP-1 in VSMCs derived from AMPKα2^−/−^ mice (Fig. 3A–C). ELISA analysis also confirmed that PA markedly increased the secretion of IL-6 and MCP-1 by VSMCs from AMPKα2^−/−^ mice when compared to secretion by VSMCs from WT mice. DHA suppressed IL-6 and MCP-1 secretion in WT VSMCs but not in those from AMPKα2^−/−^ mice (Fig. 3D, E).

### GPR120 is involved into the anti-inflammatory effect of DHA in VSMCs

**Fig. 4:**
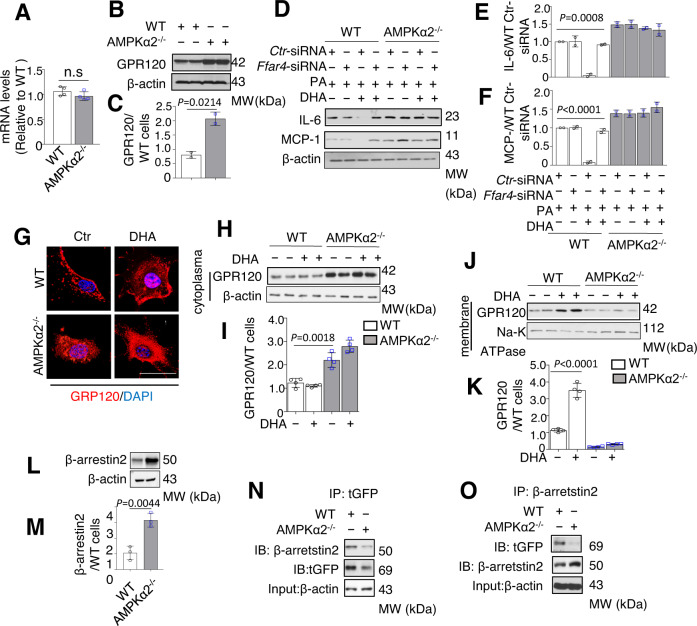
Lack of AMPKα2 blocks DHA-induced trans-localization of GPR120 to plasma membrane and interaction with β-arrestin 2. **A** Representative Quantification of GPR120 mRNA expression in WT and AMPKα2^−/−^ VSMCs (*n* = 4). **B**, **C** Western blotting and quantification of GPR120 expression in primary WT and AMPKα2^−/−^ VSMCs (*n* = 2). **D**–**F** Western blotting and quantification of IL-6 (**D**) and MCP-1 (**E**) expression in WT and AMPKα2^−/−^ VSMCs transfected with *Ctr*-siRNA or *Ffar4*-siRNA and treated with PA (300 μM) and/or DHA (50 μM). **G** Representative immunofluorescence images showing GPR120 localization in WT and AMPKα2^−/−^ VSMCs, before and after a 1-h DHA treatment. Scale bar, 25 μm. **H**–**K** Representative western blots (**H**, **J**) and quantification (**I**, **K**) of GPR120 expression in cytoplasmic and membrane fractions before and after a 1-h DHA treatment (*n* = 4). **L**, **M** Western blot and quantification of β-arrestin 2 expression in WT and AMPKα2^−/−^ VSMCs (*n* = 3). **N**, **O** IP assays for studying GPR120–β-arrestin 2 interactions in WT and AMPKα2^−/−^ VSMCs. All data shown represent one of two separate experiments. Quantification of western blots was performed using Image-Pro plus software, distinctly from loading controls. Data are presented as the mean ± s.e.m. *p* value by two-sided Student’s *t* tests. *Ctr*-siRNA control siRNA, *Ffar4*-siRNA siRNA for mouse *Ffar4*, IB immunoblot, IP immunoprecipitation.

Next, we tested if GPR120 (*Ffar4*) is required for FO-induced anti-inflammatory effects in VSMCs. As shown in Fig. 4A to C, GPR120 protein level but not mRNA level was significantly higher in AMPKα2^−/−^ VSMCs than that in WT VSMCs, indicative of an increased protein stability of GRP120. Furthermore, the levels of PA-stimulated MCP-1 and IL-6 secretion were higher in *Ffar4*-specific siRNA-transfected WT VSMCs with DHA treatment, but no changes were observed in *Ffar4*-specific siRNA-transfected AMPKα2^−/−^ VSMCs (Fig. 4D–F).

### Lack of AMPKα2 blocks the membrane trans-localization of GPR120 and interaction with β-arrestin 2

Endogenous GPR120 localizes to both the plasma membrane and cytoplasm of WT VSMCs (Fig. 4G). After stimulation with DHA for 30 min, a greater amount of GPR120 was detected at the plasma membranes of WT cells but not AMPKα2^−/−^ cells (Fig. 4H–K). To confirm this translocation, we observed green fluorescent protein-tagged GPR120 (GPR120-tGFP) over time. Under baseline (resting) conditions, GPR120-tGFP was localized both in the cytoplasm and at the membranes of WT VSMCs. DHA treatment induced GPR120-tGFP to localize from the cytoplasm to the membrane. In unstimulated AMPKα2^−/−^ VSMCs, a substantial proportion of GPR120-tGFP was localized in perinuclear regions. After treatment with DHA, GPR120-tGFP localization did not change in AMPKα2^−/−^ VSMCs. In contrast, treatment of AMPKα2^−/−^ VSMCs with ML792 significantly restored DHA-induced GPR120 membrane translocation (Fig. S4A, S4B, Supplementary Movies 1–3). β-arrestin 2 is a key down-regulated molecule of GPR120 that mediated its effects. Next, we examined if AMPKα2 deletion altered the interaction of β-arrestin2 with GPR120. The expression of β-arrestin 2 was higher in AMPKα2^−/−^ VSMCs than WT VSMCs (Fig. 4L, M). However, an IP assay revealed that the interaction of the GPR120-tGFP fusion protein with β-arrestin2 was dramatically reduced in AMPKα2^−/−^ VSMCs compared to WT VSMCs (Fig. 4N, O).

### AMPKα2 deficiency increases the GPR120 SUMOylation at K32

**Fig. 5:**
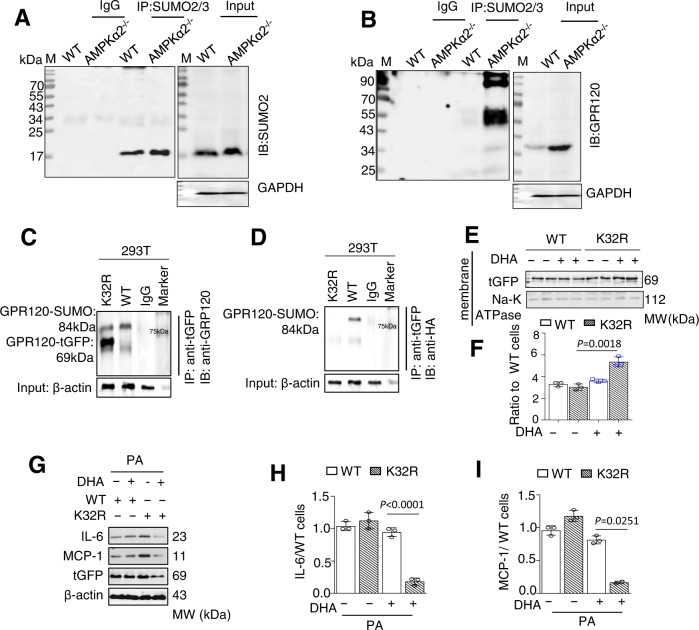
GPR120 SUMOylation at K32 blocked the effects of DHA in AMPKα2^−/−^ cells. **A**, **B** IP assays showed endogenous GPR120 and SUMOylated GPR120 in WT and AMPKα2^−/−^ VSMCs by western blotting of whole cell extracts, prepared in the presence of isopeptidase inhibitors (**C**, **D**) IP assays for whole cell extracts from 293T cells co-transfected with either the WT GPR120-tGFP or K32R/GPR120-tGFP vector and the SUMO2-HA vector. **E**, **F** Western blotting (**E**) and quantification (**F**) of tGFP expression in membrane fractions from AMPKα2^−/−^ VSMCs transfected with GPR120-tGFP or K32R/GPR120-tGFP and treated for 1 h with DHA. Scale bars, 25 μm. **G**–**I** Western blotting (**G**) and quantification of IL-6 (**H**) and MCP-1 (**I**) expression in AMPKα2^−/−^ VSMCs transfected with GPR120-tGFP or K32R/GPR120-tGFP and treated with PA (300 μM) and DHA (50 μM) for 1 h. All IP and western blotting data shown represent one of two separate experiments, distinctly from loading controls. Quantification of western blot results was performed using Image-Pro plus software. Data are presented as the mean ± s.e.m. *p* value by two-sided Student’s *t* tests. K32R lysine 32 mutated to arginine, PA palmitic acid, DHA docosahexaenoic acid.

A high probability conserved SUMOylation site was found in the N-terminal amino acid lysine 32 (K32) of GPR120 (Fig. S4C). IP and western blotting for SUMO2/3 revealed that AMPKα2^−/−^ VSMC lysates had higher levels of SUMOylated protein than WT VSMC lysates (Fig. 5A). As expected, an increased amount of SUMOylated GPR120 was found in AMPKα2^−/−^ VSMCs compared to WT VSMCs in the membrane (Fig. 5B). Further, 293T cells were transfected with both GPR120-tGFP and SUMO2-HA vectors. GPR120 was first immunoprecipitated with an antibody against tGFP. Western blotting for GPR120 revealed two bands with molecular masses of 84 kD and 69 kD (Fig. S4D). Only the 84-kD band was detected when the same gels were blotted with an antibody against the HA tag (Fig. S4E).

293T cells co-transfected with the WT plasmid exhibited a dark band for SUMOylated (84 kD) and a lighter band for non-SUMOylated (69 kD) GPR120 (Fig. 5C). By contrast, in cells transfected with K32R/GPR120-tGFP plasmids, GRP120 was predominantly non-SUMOylated, which was confirmed by blotting for the HA tag (Fig. 5D).

### Blockage of GPR120 SUMOylation can rescue the anti-inflammatory effect and restore the degradation of GPR120 protein by DHA treatment in AMPKα2^−/−^ VSMCs

After treatment with DHA for 1-h, GPR120-tGFP translocated to the membrane in the K32R mutant, while it remained largely localized to perinuclear areas in WT construct-transfected cells in AMPKα2^−/−^ VSMCs (Fig. S4F and Supplementary Movies 4–5). This observation was confirmed through quantification of membrane proteins (Fig. 5E, F). Meanwhile, an IP assay revealed that the interaction of the K32R/GPR120-tGFP fusion protein with β-arrestin 2 was dramatically increased compared to that of GPR120-tGFP (WT) in 293T cells with the SUMO2-HA plasmid (Fig. S4G). Meanwhile, exposure of AMPKα2^−/−^ VSMCs expressing WT GPR120 to PA and DHA significantly increased IL-6 and MCP-1, both levels were significantly lower in cells expressing the non-SUMOylated GPR120 mutant (Fig. 5G–I). Interestingly, we detected a significant reduction in K32R/GPR120-tGFP expression compared to WT/GPR120-tGFP in AMPKα2^−/−^ VSMCs when treated with by DHA for two hours (Fig. S4H, I). The reduction in K32R/GPR120-tGFP with DHA treatment occurs only at the protein level, not at the mRNA level (Fig. S4J). This implies that inhibition of SUMOylation restores DHA-induced degradation of GPR120.

### Lack of AMPKα2 increases the expression of UBC9 and SUMO2/3 to promote the GPR120 SUMOylation

**Fig. 6:**
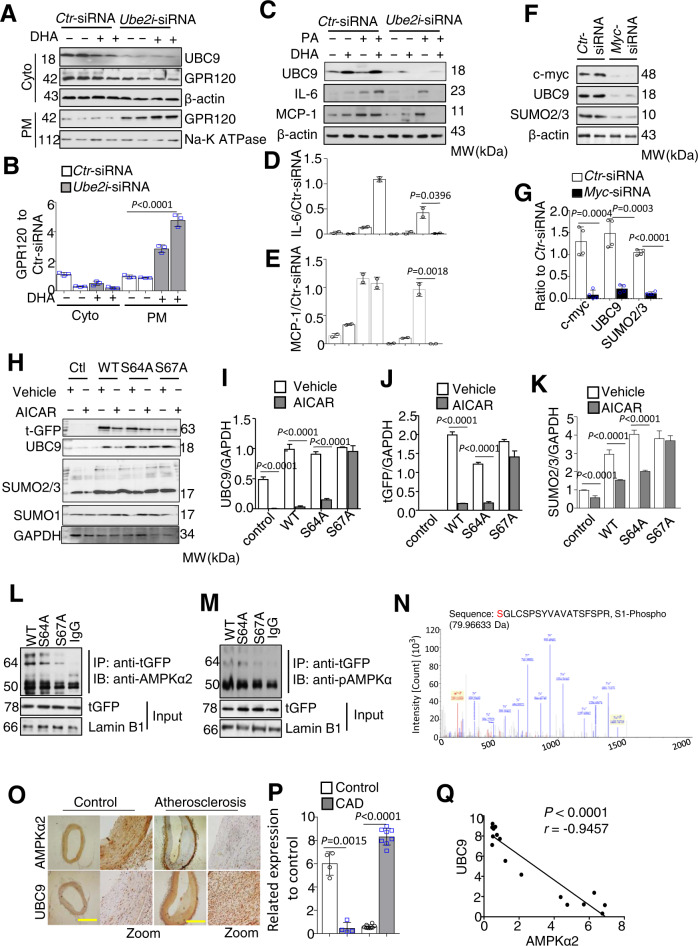
Increases in the expression of UBC9 and SUMO2/3 promotes the GPR120 SUMOylation in AMPKα2^−/−^ VSMCs by activating the C-MYC S67 phosphorylation. **A**, **B** Western blotting and quantification of UBC9 and GPR120 expression in cytoplasmic and membrane fractions from AMPKα2^−/−^ VSMCs transfected with *Ctr*-siRNA or *Ube2i*-siRNA, with or without DHA (50 μM) treatment (*n* = 4). *p* value versus *Ctr*-siRNA by one-way ANOVA tests. **C**–**E** Western blotting and quantification of UBC9, IL-6, and MCP-1 expression in AMPKα2^−/−^ VSMCs transfected with *Ctr*-siRNA or *Ube2i*-siRNA before or after PA (300 μM) and DHA (50 μM) treatment for 1 h (*n* = 2). **F**, **G** Western blotting and quantification of c-myc, UBC9, and SUMO2/3 expression in AMPKα2^−/−^ VSMCs transfected with Ctr-siRNA or *Myc*-siRNA (*n* = 4). **H**–**K** Western blotting (**H**) and quantification of tGFP (**I**), UBC9 (**J**), and SUMO2/3 (**K**) expression in 293T cells transfected with WT *C-myc* (WT), S64A/*C-myc* (S64A), or S67A/*C-myc* (S67A) vectors and treated for 2 hrs with AICAR (1 mM) (*n* = 2). **L** Extracts from 293T cells transfected with either WT, S64A, or S67A c-myc constructs were prepared to assess the interactions between the c-myc-tGFP fusion protein and AMPKα2 or pAMPKα. **M** Recombinant AMPK and recombinanted c-myc-tGFP protein were incubated in the kinase buffer with ATP. Immunoprecipitation was done using anti-tGFP antibody. **N** The phosphorylation of serine at amino acids 67 in the peptide PTPPLSPSRRSG by LC-MS/MS followed processed using Proteome Discoverer 1.3. **O** Representative images of immunostaining showing AMPKα2 and UBC9 expression and localization in human artery tissues from control (*n* = 4) and CAD patients (*n* = 8). Scale bar, 400 μM. **P** Semi-quantification analysis of immunostaining using Image-Pro plus software. Correlational analyses of AMPKα2 expression and the expression of UBC9 (**Q**) in human artery tissues. All data shown represent one of three separate experiments. Quantification of western blots was performed using Image-Pro plus software, distinctly from loading controls. Data are presented as the mean ± s.e.m. *p* value by two-sided Student’s t tests. AICAR 5-aminoimidazole-4-carboxamide 1-α-d-ribofuranoside, *Ube2i*-siRNA siRNA against mouse *Ube2i*, *Myc*-siRNA siRNA against mouse Myc, CAD coronary artery disease.

As depicted in Fig. S5A, the expression levels of SUMOylation-related genes were significantly higher in AMPKα2^−/−^ VSMCs than in WT or AMPKα1^−/−^ VSMCs. These differences were confirmed by quantifying the relative mRNA levels in VSMCs and aortic tissues by RT-PCR (Fig. S5B, C). Accordingly, analyses of protein expression revealed that the levels of UBC9 and SUMO2/3 were higher in AMPKα2^−/−^ VSMCs aortic tissues than in those from WT or AMPKα1^−/−^ mice (Fig. S5D–G). Furthermore, the amount of GPR120 in membrane fractions was significantly higher in AMPKα2^−/−^ cells in which the gene encoding UBC9 (*Ube2i*) was silenced (Fig. 6A, B). In addition, DHA lowered the PA-mediated induction of MCP-1 and IL-6 in *Ube2i*-siRNA-transfected but not in control siRNA-transfected AMPKα2^−/−^ VSMCs (Fig. 6C–E, Fig. S5H, I).

### AMPKα2 reduced the stability of C-MYC through Serine 67 phosphorylation to inhibit the expression of UBC9 and SUMO2/3

The expression levels of c-myc and S62-phosphorylated c-myc (Nuclei c-myc) in whole cell lysates and nuclei were higher both in AMPKα2^−/−^ VSMCs and aortic tissue than in their WT counterparts (Fig. S6A–F). However, the mRNA levels of c-myc were similar between WT and AMPKα2^−/−^ VSMCs (Fig. S6G). Importantly, silencing *My*c expression markedly reduced the expression of UBC9 and SUMO2/3 in AMPKα2^−/−^ VSMCs (Fig. 6F, G).

Two potential conserved AMPK phosphorylation sites were found, namely S64 and S67 (Fig. S6H, I). Compared to those in non-transfected cells, the expressions of tGFP, UBC9, and SUMO2/3 were significantly increased in 293T cells transfected with each of these *C-myc* vectors (Fig. 6H–K). However, treatment with AICAR (1 mM), a potent AMPK activator, significantly decreased the expression of these proteins in cells transfected with either WT/c-myc-tGFP or S64A/*C-myc*-tGFP but not with S67A/*C-myc*-tGFP. In addition, AMPKα2 and pAMPKα coimmunoprecipitated with the c-myc protein in nuclei from 293T cells transfected with WT/*C-myc*-tGFP or S64A/*C-myc*-tGFP but not with S67A/*C-myc*-tGFP (Fig. 6L, M). The phosphorylation of serine 67 in peptide sGLCsPSYVAVATSFSPR by AMPK was confirmed via LC-MS/MS analysis (Fig. 6N).

### Expression of UBC9 is inversely correlated with AMPKα2 in human arteries

Normal arteries exhibited strong staining for AMPKα2 and weak staining for UBC9. In contrast, atherosclerotic arteries from CAD patients exhibited significantly weaker staining for AMPKα2 and stronger staining for UBC9 (Fig. 6O, P). Correlation analyses revealed significant inverse relationships between AMPKα2 expression and that of UBC9 (Fig. 6Q).

### EPA and DHA negatively correlated with severity of CAD patients with high levels of AMPKα2 expression

**Fig. 7:**
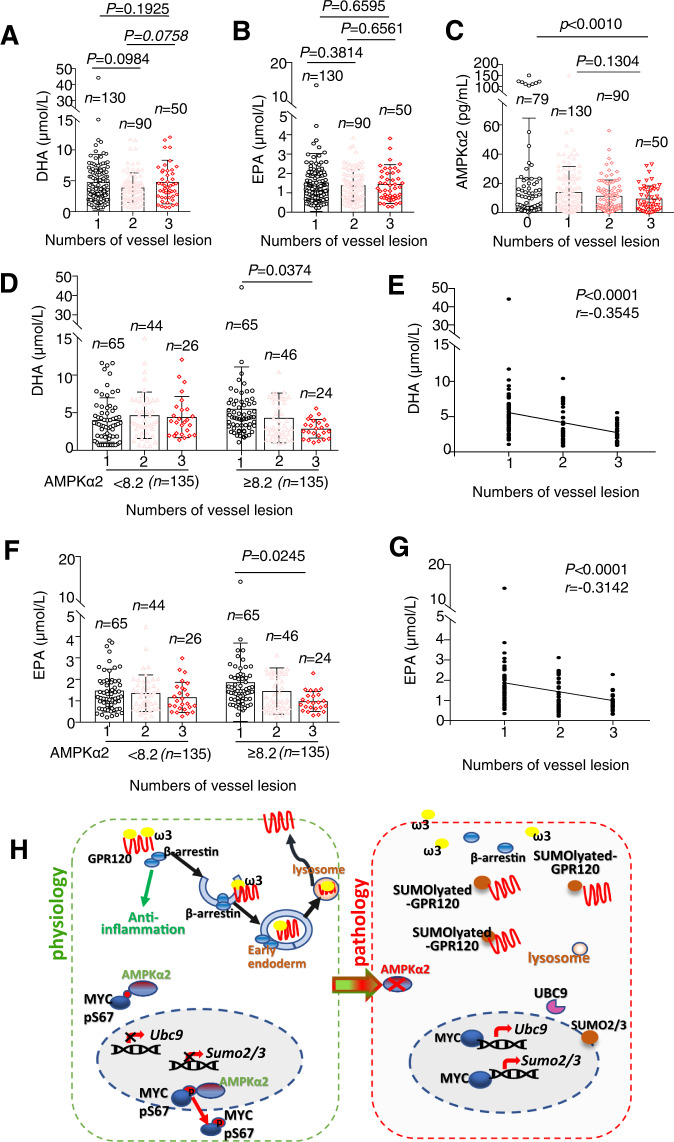
AMPKα2 expression determined the protective effect of FO in CAD patients. Plasma DHA (**A**), EPA (**B**), or AMPKα2 (**C**) in platelet versus CAD severity in 349 patients based on vessel-lesion numbers: 0 no lesion vessel represent non-CAD, 1 single vessel represents low severity, 2 two vessels represent medium severity, and 3 three vessels represent high severity. Data are presented as the median ± s.e.m. and were analyzed by one-way ANOVA with the Kruskal–Wallis test for multiple comparisons. Plasma DHA (**D**) and EPA (**F**) concentrations in CAD patients and vessel-legion numbers versus AMPKα2 activation in platelet-rich plasma. A grayscale value of <8.2 pg/mL for IP assay obtained AMPKα2 activation indicates low AMPKα2 activity and a value of ≥8.2 indicates high AMPKα2 activity. *p* value via one-way ANOVA with the Kruskal–Wallis test for multiple comparisons. Scatter plots showing correlations between plasma DHA (**E**) or EPA (**G**) concentrations in CAD patients with high AMPKα2 activation (*n* = 135). Spearman’s correlation-coefficient test was used to calculate *r* and *p* values. DHA docosahexaenoic acid, EPA eicosapentaenoic acid. **H** A Schematic figure showed that AMPKα2 mediated the anti-inflammation of FO. In physiological condition, AMPKα2 can phosphates C-MYC at 67 serine to block its translocation into the nuclei, then represses the transcription of UBC9 and SUMO2/3 and GPR120 SUMOylation. Pathologically, inactivation of AMPKα2 leads to the nuclei trans-localization of C-MYC to enhance the expression of UBC9 and SUMO2/3 and GPR120 SUMOylation in VSMCs.

To confirm the relationship between AMPKα2 and FO anti-atherosclerosis, we enrolled 349 patients (270 CAD, 79 non-CAD) from the General Hospital of Northern theater command between April 2017 and June 2019. Supplemental Table 2 shows the clinical characteristics of the patients. As expected, the plasma concentrations of DHA and EPA were significantly higher in 79 non-CAD group than in 270 CAD patients (Fig. S7A, B). However, multi-vessel lesion analysis revealed no association between plasma concentrations of EPA and DHA and the number of vessel lesions, suggesting there is no association between EPA or DHA concentration and angiographic severity of CAD (Fig. 7A, B). Through comparative analysis with AMPKα2 standard products, the expression of AMPKα2 in the platelets is semi-quantitatively used to evaluate in non-CAD patients or CAD patients. The results suggest that AMPKα2 expression is negative correlated with the severity of CAD patients’ lesions (Fig. S7C and Fig. 7C). Subgroup analysis revealed that the severity of CAD did not correlate with neither DHA nor EPA concentrations in blood in patients with lower AMPKα2 levels (AMPKα2 < 8.2 pg/mL, *n* = 135) (Fig. 7D, E). There was a significant negative correlation between the concentrations of EPA or DHA and the severity of CAD in patients with higher AMPKα2 levels (AMPKα2 ≥ 8.2 pg/mL, *n* = 135) (Fig. 7F, G).

## Discussion

In the present study, we identified that AMPKα2 activation suppresses GPR120 SUMOylation by inhibiting UBC9 and SUMO2/3 expressions. SUMOylated GPR120 loses its ability to mediate the anti-inflammatory function of FO in vitro and in vivo, which is essential for its anti-atherosclerotic effects (Fig. 7H).

AMPK is an important metabolic sensor that regulates multiple physiological processes, including lipid and glucose metabolism as well as energy homeostasis^25–27^. Besides its role in metabolic processes, AMPK was also reported to exhibit anti-inflammatory and anti-tumor effects^28,29^. Genetic deletion of AMPKα2, but not of AMPKα1, increases atherosclerosis in low-density lipoprotein receptor-deficient mice, indicative of AMPKα2’s role as a powerful negative regulator of VSMC phenotypic switching^24^. Consistent with its functions, AMPKα2 activity is reportedly suppressed in CVD, which is associated with obesity, diabetes, atherosclerosis, and hypertension^20,22–24^. In the present study, we observed an inverse correlation between FO administration and the severity of coronary artery lesions only in CVD patients with high AMPKα2 expression. Moreover, in animal models, AMPKα2 deficiency, both across all tissues or VSMC-specific, aggravates diet-induced atherosclerosis and compromises the efficacy FO. In addition to FO, AMPK activation is considered an essential step for the efficacy of metformin, rosiglitazone, and statins^30,31^.

SUMOylation is a second prominent post-translational modification of lysine residues^32,33^. Modification of proteins by SUMO1, 2, and 3 is reported to control protein function, subcellular localization, and/or expression^32^. Whether or not SUMOylation impacts CVD remained largely unknown. In the present study, we also identified the site of GPR120 SUMOylation at K32 in the N-terminal region. Previous evidence suggests that the effects of omega-3 fatty acids mediated by GPR120 are dependent on its binding to β-arrestin2^19^. The results of the present study further indicate that, while more stable, SUMOylated GPR120 fails to translocate to the membrane, bind to β-arrestin 2, and suppress inflammation. Mutations in the key K32 residue rescue FO-mediated receptor internalization and anti-inflammatory effects.

Interestingly, β-arrestin2 is known to promote SUMOylation of several proteins, including SERCA2a^34,35^. Whether β-arrestin2 is a negative feedback mechanism on SUMOylation of GPR120. To test the hypothesis, we silenced the β-arrestin 2 to detect the expression of SUMOylation proteins SUMO-2/3, UBC9, and GPR120 in WT and AMPKα2^−/−^ VSMCs. However, in AMPKα2 deficient-induced elevation of SUMOylation in VSMCs, β-arrestin 2 only mediated the binding between GPR120 and β-arrestin 2 and its downstream anti-inflammation, but not involved into the GPR120 SUMOylation.

A recent study suggested that FO consumption may prevent certain cancers and may be linked to a reduced incidence of cancer^36,37^. Our finding that AMPKα2 regulates C-MYC stability may help explain these effects as well as those of AMPK agonists, including metformin. Mechanistically, we found that AMPKα2 promotes the degradation of C-MYC by phosphorylating it at Serine 67. C-MYC is the principal member of a nonredundant family of transcription factors and is believed to regulate the expression of ~15% of all genes. A dysregulation of C-MYC leads to tumorigenesis in mouse models and is reported in almost all human cancers, which is involved in regulating a myriad of cellular processes, such as cell cycle progression, cell growth, differentiation, metabolism, and apoptosis^38,39^. A reduction of C-MYC stability via phosphorylation by AMPKα2 may, therefore, reduce the proto-oncogenic effects of C-MYC, contributing to tumor suppression or prevention. Further study is warranted to determine to test the effect of AMPKα2 anti-tumorigenesis. Since the expression and activity of AMPKα2 in the vascular tissue of CAD patients could not be evaluated, we could only use changes in the expression of AMPKα2 in the platelets of patients. The correlation between platelets and AMPKα2 expression in vascular tissues requires further verification. Controlled intervention verification of FO in people with low versus high platelet expression of AMPKα2 is also required to further verify our conclusions.

Taken together, our findings might have broad implications across physiological and pathological conditions of AMPK activation. We conclude that AMPKα2 is required to maintain the anti-inflammatory and anti-atherosclerotic effects of FO in CAD patients. Therefore, the beneficial effects of these stimuli or AMPK activators may be enhanced through the daily consumption of FO.

## Methods

### Patient information

A total of 349 patients with 270 CAD and 79 Non-CAD individuals, aged between 29 and 75 years, were enrolled in the study from April 2017 to June 2019. The patients were unrelated Han individuals from the Northern Theater Command, China. All the subjects had undergone coronary angiography for the evaluation of suspected or established CAD at General Hospital of Northern Theater Command, China. The study was approved the ethics committees of General Hospital of Northern Theater Command (K2017-16). The study was authorized, and the study design and conduct complied with all relevant regulations regarding the use of human study participants and was conducted in accordance with the criteria set by the Declaration of Helsinki. Informed consents were obtained from all participants in the study.

Subjects who had inflammatory diseases, valvular heart disease, cancers, or rheumatoid arthritis were excluded. The inclusion and exclusion criteria are listed in Supplementary Table 1, respectively. The Non-CAD patient was those with atherosclerosis lesion to artery lumen ratios of <30%. Inclusion criteria for CAD were ≥50% stenosis in ≥1 major epicardial coronary arteries as determined by percutaneous transluminal coronary angiography, with or without antecedent revascularization. The severity of CAD was evaluated by single-vessel coronary artery disease and multi-vessel coronary artery disease (defined as at least two major vessels [≥2 mm in diameter] with >70% stenosis of the diameter). Complete clinical histories were obtained from all subjects, the baseline characteristics are shown in Supplementary Table 2. The information FO consumption in the participants was first obtained from the responses to a questionnaire survey and FO levels were verified by the assays with gas chromatography analysis. Diabetes mellitus information was obtained from (1) self-reported diagnosis, (2) anti-diabetes treatment, and (3) fasting serum glucose ≥7 mmol/L. All patients received standard medical therapy per discretion of the attending cardiologists. Drug-treated hypertension, hypercholesterolemia, and hyperglycemia were identified from self-reported use of blood pressure-lowering and lipid-lowering drugs, respectively. Blood samples were collected after overnight fasting. Platelet-rich plasma and plasma samples were stored at −80 °C until analysis.

### Measurement of fish oils

All samples were detected by Bio-tech Technical Company, Wuhan, China (http://www.smi-wh.cn/). The detection method can be briefly described as follows: arachidonic acid-derived eicosanoids and five deuterium-labeled internal standards were purchased from Cayman Chemical (Ann Arbor, Michigan, USA). All other chemicals were purchased from Sigma-Aldrich (Maryland, USA). Serum samples (total volume 80 µL) and 10 μL butylated hydroxytoluene-methanol (MeOH) solution (4.8 g/100 mL) were subjected to protein precipitation by adding 130 μL of pure MeOH and 100 μL of MeOH containing deuterium-labeled internal standards with a final concentration of 50 ng/mL each of prostaglandin E2-d4, 6-keto prostaglandin F1-d4, 5(S)-HETE-d8, 9(S)-HODE-d4, and 200 ng/mL arachidonic acid-d8. The samples were centrifuged at 12,000 rpm for 10 min at 4 °C, and the supernatants were transferred into new tubes and diluted with deionised water (containing 0.005% formic acid) to 15% MeOH concentration. Solid-phase extraction was performed using Waters Oasis HLB extraction cartridges (Waters Corporation, Milford, Massachusetts, USA). The extractions were dried with a SpeedVac (SPD2010; Thermo Fisher Scientific, Waltham, MA) and dissolved in 100 μL MeOH for analysis by liquid chromatography (Agilent 1290; Agilent, San Jose, CA) coupled with electrospray ionization on a triple quadrupole mass spectrometer (Agilent 6470). For analysis, 3 μL of the extraction was injected, and the auto sampler was cooled to 4 °C. Chromatographic separation was achieved on an Agilent ZORBAX Eclipse Plus C18 column (2.1 × 100 mm, 1.8 μm) using a flow rate of 0.65 mL/min at 45 °C during a 13 min gradient- (0–12 min from 68% solution A [water containing 0.005% formic acid] to 20% A, 12–13 min 5% solution A); solvent B was acetonitrile containing 0.005% formic acid. Electrospray ionization was performed in the negative ion mode. The source parameters were as follows: drying gas (N2) flow of 10 L/min at 300 °C, nebulizer pressure of 30 psi, the sheath gas (N2) temperature was 350 °C with a flow rate of 11 L/min, the capillary was set at 3500 V, and the nozzle voltage was 500 V. Multiple reaction monitoring was used for quantifying the screening fragment ions. The peak determination and peak area integration were performed with Mass Hunter (Agilent, version B.08.00), whereas autointegration was manually inspected and corrected if necessary. The obtained peak areas of each target were corrected by the appropriate internal standards, and calculated response ratios were used throughout the analysis. Representative pictures are shown in Fig. S1A, B.

### Animal models

Low-density lipoprotein receptor knockout (LDLR^−/−^) (C57BL/6 background) mice (5–6 weeks of age) purchased from the Jackson Laboratory (Bar Harbor, ME). *Prkaa2* floxed (AMPKα2^fl/fl^) mice, LDLR^−/−^/AMPKα2^−/−^ mice, and LDLR^−/−^/AMPKα2^sm22Cre^ (VSMC-specific *Prkaa2* knockout) mice were kindly provided by Dr. Zou’s lab^24^. All male mice were fed a western diet (contains 0.21% cholesterol) for 12 weeks to induce atherosclerosis and grouped with or without 5% FO administration. The animal protocol was approved by the animal ethics committees of General Hospital of Shenyang Northern Theater Command.

### Reagents

Anti-tGFP (TA150039) was purchased from OriGene (Rockville, MD). Anti-AMPKα (#5831), anti-pAMPKα-Thr172 (#2535), anti-AMPKα1 (#2795), anti-AMPKα2 (#2757), anti-c-myc (#5605), anti-pc-myc-Ser62 (#13748), anti-UBC9 (#4786), anti-IL-6 (#13797), anti-NaK ATPase (#7074), anti-α-smooth muscle actin (SMA; #56856), anti-SUMO2/3 (#4971), and anti-MCP-1 (#12838) were purchased from Cell Signaling Technology Inc. (Danvers, MA). Anti-β-arrestin 2 (C16D9) rabbit mAb (Cell Signaling Technology) for the western blotting analysis, and sc-365445 (Santa Cruz Biotechnology) for the IP assay. Anti-β-arrestin 1 (D8O3J) rabbit mAb (Cell Signaling Technology). Anti-α-actin (sc-47778) and anti-SUMO1 (sc-5380) were purchased from Santa Cruz Biotechnology, Inc. (Dallas, TX). Anti-GPR120 (sc-390752) and anti-SUMO2/3 immunoprecipitation (IP) beads (#BK-162) were purchased from Cellskeleton, Inc and antibody (sc-50331) were purchased from Santa Cruz Biotechnology, Inc. GW9508 (G9797) and AICAR (A9978) were purchased from Sigma-Aldrich (St. Louis, MO). DHA (CAS6217-54-5) was purchased from Cayman Chemical Company (Ann Arbor, MI). Anti-CD68 (14-0681-80) was purchased from Affymetrix-ebioscience.

### Cell culture

Mouse primary VSMCs were isolated from mice as previously reported^4,5^. Briefly, VSMCs were isolated from cultured explants of aortas from 8- to 10-week-old wild-type C57BL/6J (WT), AMPKα1^−/−^, and AMPKα2^−/−^ mice.

### Western blotting and IP

Cells or aorta tissues were homogenized in RIPA lysis buffer (sc-24948; Santa Cruz Biotechnology, Inc.), and protein contents were measured using the Bradford (bicinchoninic acid) assay (#23225; Pierce Biotechnology, Rockford, IL). Immuno-precipitates or cell lysates were subjected to western blotting with specific primary antibodies followed by detection with horseradish peroxidase-conjugated secondary antibody and enhanced chemiluminescence. Target protein expression was normalized to α-actin or GAPDH to correct for loading.

### Histology and immunohistochemistry

Aortic roots were cut in 5-µm-thick serial cryosections and stained with Oil Red O to quantify the lesion sizes. For immunohistochemistry, sections were incubated first with primary antibodies (against SM α-SMA, CD68, AMPKα2, UBC9, and GPR120) and subsequently with a horseradish peroxidase-conjugated secondary antibody and diaminobenzidine (ABC kit; Vector Labs) or with fluorochrome-conjugated secondary antibodies.

### Quantitative real-time polymerase chain reaction

Total RNA was extracted from cells or aorta tissues with a RNeasy mini kit (#74106; Qiagen N.V., Germany) and reverse transcribed with an iScript cDNA synthesis kit (#170-8891; Bio-Rad Laboratories, Inc., Hercules, CA). Real‐time polymerase chain reaction (RT-PCR) was performed with the CFX96 real-time system (Bio-Rad Laboratories, Inc.). The primer sequences for mouse genes are as follows (5′-3′): *Gapdh*, (F) CTA C CCC ACG GCA AGT TCA, (R) CCA GTA GAC TCC ACG ACA AC; *Ffar4* (F) CCA TCC CCT CTA GTG CTC GTC, (R) TGC GGA AGA GTC GGT AGT CT; *Ube2i*, (F) TCA TCC AAA CGT GTA TCC TTC TG, (R) CTT GTG CTC GGA CCC TTT TCT; *Sumo2/3*, (F) CTG GGG AGG TGA CCT TAG TGA, (R) GTG ATA ATC TGG ACG ATA GGC TG; *Myc*, (F) GCC ACC ACC AGC AGC GAC TC, (R) GGG GGG TGC GGC GTA GTT GTG. Target gene expression was normalized to *Gapdh*, and the fold induction was calculated with the comparative ^Δ^CT method and presented as a relative transcript level (2^−ΔΔ^CT).

### Plasmid construction and transfection

The tGFP-GPR120 (MRG208211) plasmid was purchased from OriGene (Rockville, MD). The K32R/GFP120-tGFP, mouse WT/c-myc-tGFP, S64A/c-myc-tGFP (from serine to alanine), and S67A/c-myc-tGFP plasmids were constructed in our lab and sequenced by Takara Bio Inc. (Kusatsu, Japan). Lipofectamine 2000 (11668-019; Life Technologies, Carlsbad, CA) or primary cell Nucleofector (V4XP-3012; Lonza Inc., Allendale, NJ) kits were used for plasmid transfection in mouse primary VSMCs, BMDM, human umbilical vein endothelial cells (HUVECs), and HEK293T cells according to the instructions provided by the supplier.

### Adenovirus infection

Primary VSMCs were infected with adenovirus encoding constitutively active and dominant negative AMPKα-CA and AMPKα-DN, respectively, in normal culture medium for 48 h. An adenoviral vector encoding β-galactosidase (000197A; Applied Biological Materials Inc., Richmond, Canada) was used as a control.

### Gene silencing

Small interfering RNAs (siRNAs) targeting mouse AMPKα2 (*Pkra2*) (sc-38924), Gpr120 (*Ffar4*) (sc-607380), Ubc9 (*Ube2i*) (sc-36774), and c-myc (*Myc*) (sc-29227) were purchased from Santa Cruz Biotechnology, Inc. Mouse primary VSMCs were transfected with 10 μM siRNA using Lipofectamine RNA iMAX (13778150; Life Technologies) according to the manufacturer’s instructions.

### Immunofluorescence and time-lapse imaging

Cells were fixed with 3.7% formaldehyde (v/v) in PBS and permeabilized with 0.2% Triton X-100 (v/v) in PBS for 15 min each at room temperature. The cells were blocked with 5% normal goat serum (BioGenex, Fremont, CA) for 30 min at room temperature and then incubated first with primary antibodies (1:200; anti-GPR120) at 37 °C for 30 min and then with Alexa Fluor 488- or 647-conjugated secondary antibodies (1:50; Life Technologies) at 37 °C for 45 min. Images were captured using a confocal microscope (LSM800; Carl Zeiss Microscopy Ltd, Cambridge, MA).

### LC-MS/MS analysis of the phosphorylation site of C-MYC

The co-tranfection of c-myc-GFP vector and AMPKα2-CA adenovirus into 293T cells. Collected cell lysates after transfected 48 h. IP pull-down the c-myc-GFP protein and isolated it using PAGE gel electrophoresis with Coomassie Brilliant Blue staining according to molecular weight (75 kDa and 90 kDa), 75 kDa contained the endogenous c-myc protein, and 90 kDa contained c-myc-GFP fusion protein (every group *n* = 1). The peptides were dissolved in-gel tryptic digestion and dried to completion and resuspended in 2% acetonitrile/0.1% formic acid, then were subjected to NSI source followed by tandem mass spectrometry (MS/MS) in Q ExactiveTM Plus (Thermo) coupled online to the UPLC. The detailed protocol as followed:

1. In-gel digestion. For in-gel tryptic digestion, gel pieces were destained in 50 mM NH_4_HCO_3_ in 50% acetonitrile (v/v) until clear. Gel pieces were dehydrated with 100 μl of 100% acetonitrile for 5 min, the liquid removed, and the gel pieces rehydrated in 10 mM dithiothreitol and incubated at 56 °C for 60 min. Gel pieces were again dehydrated in 100% acetonitrile, liquid was removed and gel pieces were rehydrated with 55 mM iodoacetamide. Samples were incubated at room temperature, in the dark for 45 min. Gel pieces were washed with 50 mM NH_4_HCO_3_ and dehydrated with 100% acetonitrile. Gel pieces were rehydrated with 10 ng/μl trypsin resuspended in 50 mM NH_4_HCO_3_ on ice for 1 h. Excess liquid was removed, and gel pieces were digested with trypsin at 37 °C overnight. Peptides were extracted with 50% acetonitrile/5% formic acid, followed by 100% acetonitrile. Peptides were dried to completion and resuspended in 2% acetonitrile/0.1% formic acid.

2. LC-MS/MS analysis. The tryptic peptides were dissolved in 0.1% formic acid (solvent A), directly loaded onto a home-made reversed-phase analytical column (15-cm length, 75 μm i.d.). The gradient was comprised of an increase from 6% to 23% solvent B (0.1% formic acid in 98% acetonitrile) over 16 min, 23% to 35% in 8 min and climbing to 80% in 3 min then holding at 80% for the last 3 min, all at a constant flow rate of 400 nl/min on an EASY-nLC 1000 UPLC system. The peptides were subjected to NSI source followed by tandem mass spectrometry (MS/MS) in Q ExactiveTM Plus (Thermo) coupled online to the UPLC. The electrospray voltage applied was 2.0 kV. The *m*/*z* scan range was 350 to 1800 for full scan, and intact peptides were detected in the Orbitrap at a resolution of 70,000. Peptides were then selected for MS/MS using NCE setting as 28 and the fragments were detected in the Orbitrap at a resolution of 17,500. A data-dependent procedure that alternated between one MS scan followed by 20 MS/MS scans with 15.0 s dynamic exclusion. Automatic gain control (AGC) was set at 5E4.

3. Data processing. The resulting MS/MS data were processed using Proteome Discoverer 1.3. The search parameters are set as follows: the database is set to the target protein sequence provided by the customer; the digestion mode is set to Trypsin/P; the number of missed cut bits is set to 2; the tolerance of primary parent ion mass error is set to 10 ppm; the mass error tolerance of secondary fragment ions is set to 0.02 Da; carbamidomethyl on Cys were specified as fixed modification. Variable modification is set to phosphorylation of serine, threonine and tyrosine, oxidation of methionine, and acetylation of N-terminal protein. Peptide confidence was set at high, and peptide ion score was set >20.

### Statistical analyses

Statistical analyses were performed with GraphPad Prism 5 (GraphPad Software, Inc., La Jolla, CA) or R version 3.1.1 (http://www.rproject.org/). Mean and standard deviation of FA proportions, blood lipids, blood glucose, blood pressure, and body mass index were calculated separately for the non-CAD Control and CAD Patients. For variables with normal distribution were evaluated by a two-tailed Student t test. Relationships between the selected serum PUFAs (EPA, DHA, LA, and ALA) were investigated by calculating the Spearman correlation coefficients. In the adjusted models, body mass index, smoking, physical activity, education, alcohol intake, diabetes mellitus, drug-treated hypertension, and drug-treated hypercholesterolemia at baseline were included as covariates. Serum FAs (EPA and DHA) were investigated as continuous (per 1-SD increase) and categorical (quartiles) variables. For comparisons of AMPKα2 expressions in human platelet-rich plasma samples, log-transformed data and the nonparametric Mann–Whitney U test were used. Pearson or nonparametric Spearman correlation coefficients were calculated for gene associations based on D’Agostino-Pearson omnibus normality test results. For mouse data, the Student’s t test, Mann–Whitney U test, or a one-way analysis of variance (ANOVA) for multiple comparisons. For two independent factors, a two-way ANOVA was used followed by Bonferroni’s post hoc tests. Possible outliers in the data sets were detected with robust regression and removed at a Q level of 5%. Differences were considered significant at a *p* value of <0.05.

### Reporting summary

Further information on research design is available in the Nature Portfolio Reporting Summary linked to this article.

## Supplementary information

Supplementary Information

Description of Additional Supplementary Files

Supplementary Movie 1

Supplementary Movie 2

Supplementary Movie 3

Supplementary Movie 4

Supplementary Movie 5

Reporting Summary

## Data Availability

All data supporting the findings described in this manuscript are available in the article and in the Supplementary Information files, and from the corresponding author upon request without any restrictions. Source data are provided with this paper.

## Source data

Source Data
