# Supplementary material for: AMPKα2 controls the anti-atherosclerotic effects of fish oils by modulating the SUMOylation of GPR120
Source: Nat Commun. 2022 Dec 13;13:7721. doi: 10.1038/s41467-022-34996-x (PMC9747961; doi:10.1038/s41467-022-34996-x)
Supplement: Supplementary file 1 — Supplementary Information [file 41467_2022_34996_MOESM1_ESM.pdf]

SUPPLEMENTARY INFORMATION

AMP-activated protein kinase α2 controls the anti-atherosclerotic effects of FO by modulation of the G protein-coupled receptor 120 SUMOylation

Cheng-hui Yan, Hai-Wei Liu, Xiao-xiang Tian, Jiayin Li, Ye Ding, Yi Li, Zhu Mei, Ming-Hui Zou, Ya-ling Han

Materials and Methods

Patient information

A total of 349 patients with 270 CAD and 79 Non-CAD individuals, aged between 29 and 75 years, were enrolled in the study from April 2017 to June 2019. The patients were unrelated Han individuals from the General Hospital of China. All the subjects had undergone coronary angiography for the evaluation of suspected or established CAD at General Hospital of Shenyang Northern Theater Command, china**.** Subjects who had inflammatory diseases, valvular heart disease, cancers, or rheumatoid arthritis were excluded. The inclusion and exclusion criteria are listed in Supplementary Table 1, respectively. The Non-CAD patient was those with atherosclerosis lesion to artery lumen ratios of <30%. Inclusion criteria for CAD were ≥50% stenosis in ≥1 major epicardial coronary arteries as determined by percutaneous transluminal coronary angiography, with or without antecedent revascularization. The severity of CAD was evaluated by single-vessel coronary artery disease and multivessel coronary artery disease (defined as at least two major vessels [≥2 mm in diameter] with >70% stenosis of the diameter). The study was approved by the Regional Committee for Medical Research Ethics in the Northeast Region Hospital of China(K2017-16). The study is complied with the declaration of Helsinki and was approved the ethics committees of the General Hospital of Shenyang Northern Theater Command. Informed consents were obtained from all participants in the study. Complete clinical histories were obtained from all subjects, the baseline characteristics are shown in Supplementary Table 2. The information FO consumption in the participants was first obtained from the responses to a questionnaire survey and FO levels were verified by the assays with gas chromatography analysis. Diabetes mellitus information was obtained from 1) self-reported diagnosis, 2) anti-diabetes

treatment, and 3) fasting serum glucose ≥7 mmol/L. All patients received standard medical therapy per discretion of the attending cardiologists. Drug-treated hypertension, hyper-cholesterolemia and hyperglycemia were identified from self-reported use of blood pressure-lowering and lipid-lowering drugs, respectively. Blood samples were collected after overnight fasting. Platelet-rich plasma and plasma samples were stored at -80°C until analysis.

Preparation of human platelets

Human platelets were isolated as described [1]. Blood from Non-CAD and CAD patients was collected in syringes containing ACD/3.8% sodium citrate (2:1) and centrifuged at 200g for 10 min. The platelet-rich plasma (PRP) was washed with modified Tyrode's-HEPES buffer (137 mmol/L NaCl, 2.8 mmol/L KCl, 12 mmol/L NaHCO3, 5 mmol/L glucose, 0.4 mmol/L Na2HPO4, 10 mmol/L HEPES, 0.1%bovine serum albumin, pH 6.5). After centrifugation at 900g for 10 min and the removal of the supernatant, the platelet pellet was resuspended in Tyrode's-HEPES buffer (pH 7.4, supplemented with 1 mmol/L CaCl2).

AMPKα2 expression in platelets protein

Platelets were homogenized in RIPA lysis buffer (sc-24948; Santa Cruz Biotechnology, Inc.), and protein contents were measured using the Bradford (bicinchoninic acid) assay (#23225; Pierce Biotechnology, Rockford, IL). Western blot with specific first antibodies AMPKα2 (#2757) followed by detection with specific pAMPKα (#2752) and horseradish peroxidase-conjugated secondary antibodies and enhanced chemiluminescence. Total protein expression was normalized to β-actin to correct for loading. Recombinant AMPKα2 (ab159189, Abcam) as standard protein to quantification the AMPKα2 expression in platelets protein in patients.

Detection of AMPKα2 phosphorylation in platelets protein

For AMPKα2 activation, AMPKα2 was immunoprecipitated from platelet lysates (2 mg protein) and was blotted with the antibody against phosphorylated AMPK (Thr172) (#2752). Quantification of gray-scale value of immunoblot using Image-Pro plus software.

Measurement of fish oils

All samples were detected by Bio-tech Technical Company, Wuhan, China. The detection method can be briefly described as follows: arachidonic acid-derived eicosanoids and five deuterium-labeled internal standards were purchased from Cayman Chemical (Ann Arbor, Michigan, USA). All other chemicals were purchased from Sigma-Aldrich (Maryland, USA). Serum samples (total volume 80 μL) and 10 μL butylated hydroxytoluene-methanol (MeOH) solution (4.8 g/100 mL) were subjected to protein precipitation by adding 130 μL of pure MeOH and 100 μL of MeOH containing deuterium-labeled internal standards with a final concentration of 50 ng/mL each of prostaglandin E2-d4, 6-keto prostaglandin F1-d4, 5(S)-HETE-d8, 9(S)-HODE-d4, and 200 ng/mL arachidonic acid-d8. The samples were centrifuged at 12,000 rpm for 10 min at 4 °C, and the supernatants were transferred into new tubes and diluted with deionised water (containing 0.005% formic acid) to 15% MeOH concentration. Solid-phase extraction was performed using Waters Oasis HLB extraction cartridges (Waters Corporation, Milford, Massachusetts, USA). The extractions were dried with a SpeedVac (SPD2010; Thermo Fisher Scientific, Waltham, MA) and dissolved in 100 μL MeOH for analysis by liquid chromatography (Agilent 1290; Agilent, San Jose, CA) coupled with electrospray ionization on a triple quadrupole mass spectrometer (Agilent 6470). For analysis, 3 μL of the extraction was injected, and the auto sampler was cooled to 4 °C. Chromatographic separation was achieved on an Agilent ZORBAX Eclipse Plus C18 column (2.1 × 100 mm, 1.8 μm) using a flow rate of 0.65 mL/min at 45 °C during a 13 min gradient-

(0-12 min from 68% solution A [water containing 0.005% formic acid] to 20% A, 12-13 min 5% solution A); solvent B was acetonitrile containing 0.005% formic acid. Electrospray ionization was performed in the negative ion mode. The source parameters were as follows: drying gas (N2) flow of 10 L/min at 300 °C, nebulizer pressure of 30 psi, the sheath gas (N2) temperature was 350 °C with a flow rate of 11 L/min, the capillary was set at 3,500 V, and the nozzle voltage was 500 V. Multiple reaction monitoring was used for quantifying the screening fragment ions. The peak determination and peak area integration were performed with Mass Hunter (Agilent, version B.08.00), whereas autointegration was manually inspected and corrected if necessary. The obtained peak areas of each target was corrected by the appropriate internal standards, and calculated response ratios were used throughout the analysis. Representative pictures are shown in Figure S1A-B.

Animals

Generation of *LDLR$^{-/-}$ AMPKα2$^{-/-}$* double knockout mice

Low-density lipoprotein receptor knockout (LDLR$^{-/-}$) (C57BL/6 background) mice (5-6 weeks of age) were purchased from the Jackson Laboratory (Bar Harbor, ME). AMPKα1$^{-/-}$ and AMPKα2$^{-/-}$ mice were generated[2] and backcrossed to a C57BL/6 background for at least ten generations. AMPKα2 floxed (AMPKα2$^{fl/fl}$) mice were kindly provided by Dr. Benoit Viollet. LDLR$^{-/-}$/AMPKα2$^{-/-}$ mice were generated by crossing AMPKα2$^{-/-}$ mice with LDLR$^{-/-}$ mice. Adult C57BL/6J (Jackson Laboratory) and AMPKα2$^{-/-}$ mice were used to obtain vascular smooth muscle cells (VSMCs) and bone marrow-derived macrophages (BMDMs). Mice were housed in a specific-pathogen-free facility on a 12-h light/dark cycle and allowed to acclimate for 1-2 weeks.

Male mice with an LDLR$^{-/-}$ background were fed a Western diet (contains 0.21% cholesterol) for 12 weeks to induce atherosclerosis [3]. To determine the role of FO in atherosclerosis, the mice were grouped as follows: LDLR$^{-/-}$ mice fed a western diet (n = 9), LDLR$^{-/-}$ mice fed a western diet with 5% FO (n = 15),

LDLR−/−/AMPKα2−/− mice fed a western diet (n = 16), and LDLR−/−/AMPKα2−/− mice fed a western diet with 5% FO (n = 18). All western diet or western diet with 5% EPA and DHA from Medallion labs (MN, 55427).

Generation of *LDLR−/− AMPKα2−/−* double knockout mice

SM22cre mice were obtained from Jackson Laboratories. AMPKα2$^{floxed/floxed}$ mice were provided by Dr. Benoit Viollet. LDLR$^{-/-}$AMPKα2^{sm22Cre} (VSMC-specific AMPKα2 knockout) mice were generated by crossing AMPKα2$^{floxed/floxed}$ mice with SM22Cre transgenic mice, then breeding into LDLR$^{-/-}$background. LDLR$^{-/-}$AMPKα2$^{flox/flox}$ mice served as controls. The animal protocol was reviewed and approved by General Hospital of Shenyang Northern Theater Command Institute Animal Care and Use Committee.

Measurement of serum cholesterol and triglyceride levels

Serum cholesterol and triglyceride levels were measured enzymatically, using Infinity reagents from Thermo DMA, according to the manufacturer's instructions.

Measurement of cytokines

Serum levels of IL-6 and MCP-1 in LDLR$^{-/-}$ and LDLR$^{-/-}$/AMPKα2$^{-/-}$ mice, which were fed a Western diet with or without FO, were measured using the enzyme-linked immunosorbent assay kit (ELISA kit, BioLegend), following the manufacturers protocol. Meanwhile, cellular supernatant IL-6 and MCP-1 in primary cultured cell were detected using ELISA kit, following the manufacturer's protocol.

Platelet and WBC counts in the whole blood of mice

Blood was collected from the common carotid artery of mice and anti-coagulated with 1/10 volume of tripotassium EDTA. After mixing, platelets and White blood cells (WBCs) were counted using a hematology analyzer (Sysmex, Japan).

Reagents

Anti-tGFP (TA150039) was purchased from OriGene (Rockville, MD). Anti-AMPKα (#5831), anti-pAMPKα-Thr172 (#2535), anti-AMPKα1 (#2795), anti-AMPKα2 (#2757), anti-c-myc (#5605), anti-pc-myc-Ser62 (#13748), anti-UBC9 (#4786), anti-IL-6 (#13797), anti-NaK ATPase (#7074), anti-α-smooth muscle actin (SMA; #56856), anti-SUMO2/3 (# ?) and anti-MCP-1 (#12838) were purchased from Cell Signaling Technology Inc. (Danvers, MA). Anti-β-arrestin 2 (C16D9) rabbit mAb (Cell Signaling Technology) for the western blotting analysis, and sc-365445 (Santa Cruz Biotechnology) for the IP assay. Anti-β-arrestin 1 (D8O3J) rabbit mAb (Cell Signaling Technology). Anti-β-actin (sc-47778), and anti-SUMO1 (sc-5380) were purchased from Santa Cruz Biotechnology, Inc. (Dallas, TX). anti-GPR120 (sc-390752), Anti-SUMO2/3 immunoprecipitation (IP) beads (#BK-162) were purchased from Cellskeleton, Inc and antibody (sc-50331) were purchased from Santa Cruz Biotechnology, Inc. GW9508 (G9797) and AICAR (A9978) were purchased from Sigma-Aldrich (St. Louis, MO). DHA (CAS6217-54-5) was purchased from Cayman Chemical Company (Ann Arbor, MI). Anti-CD68 (14-0681-80) were purchased from Affymetrix-ebioscience.

Cell culture

Mouse primary VSMCs were isolated from mice as previously reported [4, 5]. Briefly, VSMCs were isolated from cultured explants of aortas from 8- to 10-week-old wild-type C57BL/6J (WT), AMPKα1$^{-/-}$, and AMPKα2$^{-/-}$ mice.

Western blotting and IP

Cells or aorta tissues were homogenized in RIPA lysis buffer (sc-24948; Santa Cruz Biotechnology, Inc.), and protein contents were measured using the Bradford (bicinchoninic acid) assay (#23225; Pierce Biotechnology, Rockford, IL). Immuno-precipitates or cell lysates were subjected to Western blotting with specific primary antibodies followed by detection with horseradish peroxidase-conjugated secondary antibodies and enhanced chemiluminescence. Target protein expression was normalized to β-actin or GAPDH to correct for loading.

Histology and immunohistochemistry

Aortic roots were cut in 5-μm-thick serial cryosections and stained with Oil Red O to quantify the lesion sizes. For immunohistochemistry, sections were incubated first with primary antibodies (against SM α-SMA, CD68, AMPKα2, UBC9, and GPR120) and subsequently with a horseradish peroxidase-conjugated secondary antibody and diaminobenzidine (ABC kit; Vector Labs) or with fluorochrome-conjugated secondary antibodies.

Quantitative real-time polymerase chain reaction

Total RNA was extracted from cells or aorta tissues with a RNeasy mini kit (#74106; Qiagen N.V., Germany) and reverse transcribed with an iScript cDNA synthesis kit (#170-8891; Bio-Rad Laboratories, Inc., Hercules, CA). Real-time polymerase chain reaction (RT-PCR) was performed with the CFX96 real-time system (Bio-Rad Laboratories, Inc.). The primer sequences for mouse genes are as follows (5′-3′): *Gapdh*, (F) CTA C CCC ACG GCA AGT TCA, (R) CCA GTA GAC TCC ACG ACA AC; *Ffar4* (F) CCA TCC CCT CTA GTG CTC GTC, (R) TGC GGA AGA GTC GGT AGT CT; *Ube2i*, (F) TCA TCC AAA CGT GTA TCC TTC TG, (R) CTT GTG CTC GGA CCC TTT TCT; *Sumo2/3*, (F) CTG GGG AGG TGA CCT TAG TGA, (R) GTG ATA ATC TGG ACG ATA GGC TG; *Myc*, (F) GCC ACC ACC AGC AGC GAC TC, (R) GGG GGG TGC GGC GTA GTT GTG. Target gene expression was normalized to Gapdh, and the fold induction was calculated with the comparative ΔC_T method and presented as a relative transcript level ($2^{-\Delta\Delta C_T}$).

Plasmid construction and transfection

The tGFP-GPR120 (MRG208211) plasmid was purchased from OriGene (Rockville, MD). The K32R/GFP120-tGFP, mouse WT/c-myc-tGFP, S64A/c-myc-tGFP (from serine to alanine), and S67A/c-myc-tGFP plasmids were constructed in our lab and sequenced by Takara Bio Inc. (Kusatsu, Japan). Lipofectamine

2000 (11668-019; Life Technologies, Carlsbad, CA) or primary cell Nucleofector (V4XP-3012; Lonza Inc., Allendale, NJ) kits were used for plasmid transfection in mouse primary VSMCs, BMDM, human umbilical vein endothelial cells (HUVECs), and HEK293T cells according to the instructions provided by the supplier.

Adenovirus infection

Primary VSMCs were infected with adenovirus encoding constitutively active and dominant negative AMPKα-CA and AMPKα-DN, respectively, in normal culture medium for 48 hrs. An adenoviral vector encoding β-galactosidase (000197A; Applied Biological Materials Inc., Richmond, Canada) was used as a control.

Gene silencing

Small interfering RNAs (siRNAs) targeting mouse AMPKα2(sc-38924), Gpr120 (*Ffar4*) (sc-607380), Ubc9 (*Ube2i*) (sc-36774), and c-myc (*Myc*) (sc-29227) were purchased from Santa Cruz Biotechnology, Inc. Mouse primary VSMCs were transfected with 10 μM siRNA using Lipofectamine RNA iMAX (13778150; Life Technologies) according to the manufacturer's instructions.

Immunofluorescence and time-lapse imaging

Cells were fixed with 3.7% formaldehyde (v/v) in PBS and permeabilized with 0.2% Triton X-100 (vol/vol) in PBS for 15 min each at room temperature. The cells were blocked with 5% normal goat serum (BioGenex, Fremont, CA) for 30 min at room temperature and then incubated first with primary antibodies (1:200; anti-GPR120) at 37°C for 30 min and then with Alexa Fluor 488- or 647-conjugated secondary antibodies (1: 50; Life Technologies) at 37°C for 45 min. Images were captured using a confocal microscope (LSM800; Carl Zeiss Microscopy Ltd, Cambridge, MA).

LC-MS/MS analysis the phosphorylation site of C-MYC

The co-tranfection of c-myc-GFP vector and AMPKα2-CA adenovirus into 293T cells. Collected cell lysates after transfected 48 hrs. IP pull-down the c-myc-GFP protein and isolated it using PAGE gel electrophoresis with Coomassie Brilliant Blue staining according to molecular weight (75kDa and 90kDa), 75kDa contained the endogenous c-myc protein, and 90kDa contained c-myc-GFP fusion protein (every group n=1). The peptides were dissolved in-gel tryptic digestion and dried to completion and resuspended in 2% acetonitrile/0.1% formic acid, then were subjected to NSI source followed by tandem mass spectrometry (MS/MS) in Q ExactiveTM Plus (Thermo) coupled online to the UPLC. The detail protocol as followed:

1. In-gel Digestion

For in-gel tryptic digestion, gel pieces were destained in 50 mM NH4HCO3 in 50% acetonitrile (v/v) until clear. Gel pieces were dehydrated with 100 μl of 100% acetonitrile for 5 min, the liquid removed, and the gel pieces rehydrated in 10 mM dithiothreitol and incubated at 56 °C for 60 min. Gel pieces were again dehydrated in 100% acetonitrile, liquid was removed and gel pieces were rehydrated with 55 mM iodoacetamide. Samples were incubated at room temperature, in the dark for 45 min. Gel pieces were washed with 50 mM NH4HCO3 and dehydrated with 100% acetonitrile. Gel pieces were rehydrated with 10 ng/μl trypsin resuspended in 50 mM NH4HCO3 on ice for 1 h. Excess liquid was removed, and gel pieces were digested with trypsin at 37 °C overnight. Peptides were extracted with 50% acetonitrile /5% formic acid, followed by 100% acetonitrile. Peptides were dried to completion and resuspended in 2% acetonitrile/0.1% formic acid.

2. LC-MS/MS Analysis

The tryptic peptides were dissolved in 0.1% formic acid (solvent A), directly loaded onto a home-made reversed-phase analytical column (15-cm length, 75 μm i.d.). The gradient was comprised of an increase from 6% to 23% solvent B (0.1% formic acid in 98% acetonitrile) over 16 min, 23% to 35% in 8 min and climbing to 80% in 3 min then holding at 80% for the last 3 min, all at a constant flow rate of

400 nl/min on an EASY-nLC 1000 UPLC system. The peptides were subjected to NSI source followed by tandem mass spectrometry (MS/MS) in Q ExactiveTM Plus (Thermo) coupled online to the UPLC. The electrospray voltage applied was 2.0 kV. The m/z scan range was 350 to 1800 for full scan, and intact peptides were detected in the Orbitrap at a resolution of 70,000. Peptides were then selected for MS/MS using NCE setting as 28 and the fragments were detected in the Orbitrap at a resolution of 17,500. A data-dependent procedure that alternated between one MS scan followed by 20 MS/MS scans with 15.0s dynamic exclusion. Automatic gain control (AGC) was set at 5E4.

3. Data Processing

The resulting MS/MS data were processed using Proteome Discoverer 1.3. Tandem mass spectra were searched against XXX database. Trypsin/P (or other enzymes if any) was specified as cleavage enzyme allowing up to 2 missing cleavages. Mass error was set to 10 ppm for precursor ions and 0.02 Da for fragment ions. Carbamidomethyl on Cys were specified as fixed modification and oxidation on Met and XXX modification were specified as variable modifications. Peptide confidence was set at high, and peptide ion score was set > 20.

Statistical analyses

Statistical analyses were performed with GraphPad Prism 5 (GraphPad Software, Inc., La Jolla, CA) or R version 3.1.1 (http://www.rproject.org/). Mean and standard deviation of FA proportions, blood lipids, blood glucose, blood pressure, and body mass index were calculated separately for the non-CAD Control and CAD Patients. For variables with normal distribution were evaluated by a 2-tailed Student t test. Relationships between the selected serum PUFAs (EPA, DHA, LA, and ALA) were investigated by calculating the Spearman correlation coefficients. In the adjusted models, body mass index, smoking, physical activity, education, alcohol intake, diabetes mellitus, drug-treated hypertension, and drug-treated hypercholesterolemia at baseline were included as covariates. Serum FAs (EPA and DHA) were investigated as continuous (per 1-SD increase) and categorical (quartiles) variables.

For comparisons of AMPKα2 expressions in human platelet-rich plasma samples, log-transformed data and the nonparametric Mann-Whitney *U* test were used. Pearson or nonparametric Spearman correlation coefficients were calculated for gene associations based on D'Agostino-Pearson omnibus normality test results. For mouse data, the Student's *t* test, Mann-Whitney *U* test, or a one-way analysis of variance (ANOVA) for multiple comparisons. For two independent factors, a two-way ANOVA was used followed by Bonferroni's *post hoc* tests. Possible outliers in the data sets were detected with robust regression and removed at a Q level of 5%. Differences were considered significant at a p value of <0.05.

Supplementary Tables and Figures

Supplementary Table 1

Inclusion criteria	Exclusion criteria
1. CAD patients were ≥50% stenosis in ≥1 major epicardial coronary arteries as determined by percutaneous transluminal coronary angiography, with or without antecedent revascularization.	1. Severe (NYHA class IV) HF
2. The control patients were those with atherosclerosis lesion to artery lumen ratios of <30% as determined by percutaneous transluminal coronary angiography.	2. Any life-threatening disease expected to result in death within the next 2 years (other than CVD)
	3. Diagnosis or laboratory evidence of active severe liver disease
	4. Known familial lipoprotein lipase deficiency (Fredrickson type I), apoCII deficiency, or familial dysbeta lipoproteinemia (Fredrickson type III)
	5. Participation in another clinical trial involving an investigational agent within 90 days prior to screening
	6. History of acute or chronic pancreatitis
	7. Malabsorption syndrome and/or chronic diarrhea
	8.Known AIDS (HIV-positive patients without AIDS are allowed
	9. Drug or alcohol abuse within the past 6 months, and inability/unwillingness to abstain from drug abuse and excessive alcohol consumption during the study
	10. Mental/psychological impairment or any other reason to expect patient difficulty in complying with the requirements of the study or under-standing the goal and potential risks of participating in the study

Supplementary Table 1. The inclusion and exclusion criteria are listed.

Supplementary Table 2

Patient characteristic	Non-CAD (n = 79)	CAD (n = 270)	P value
Age-year	59.3 ±10.2	61.2 ± 10.8	P = 0.285
Female numbers-no (%)	19 (24.1)	38 (14.1)	P < 0.05
Male numbers-no (%)	60 (75.9)	232 (85.9)	P < 0.05
Body-mass index	28.5 ± 5.3	29.2 ± 5.5	P = 0.545
Cardiovascular history or risk factors-no (%)			
Myocardial infarction, stroke or revascularization	0 (0)	43 (25.1)	P < 0.0001
Hypertension	16(32.7)	81 (47.3)	P = 0.067
Diabetes mellitus	5 (10.2)	52 (30.4)	P = 0.004
Current smoking	19 (38.7)	101 (59.1)	P = 0.012
Cholesterol-mol/L			
Total	4.32 ± 1.22	4.19 ± 1.17	P = 0.531
High-density lipoprotein-mmol/L	1.01 ± 0.27	0.93 ± 0.25	P = 0.090
Low-density lipoprotein-mmol/L	2.41 ± 0.75	2.87 ± 3.08	P = 0.084
Triglyceride-mmol/L	2.14 ±2.70	2.07 ±2.07	P = 0.887
Fasting glucose-mmol/L	6.15 ± 2.80	6.45 ± 1.82	P = 0.580
hsCRP-mg/L	2.54±0.6	3.09±1.28	P < 0.001
Medications-no(%)			
Anti-platelet	49(100)	171(100)	P = 1.000
Anti-hypertension	12(20.3)	56(32.7)	P = 0.270
Statin	35(71.4)	125(73.1)	P =0.8534
Anti-hyperglycemia	10(17.86)	46(26.9)	P =0.585

Supplementary Table 2. Baseline Characteristics of the non-CAD Control (79) and CAD Patients (270). Percutaneous transluminal coronary angiography was performed in all individuals. The criteria for CAD were ≥50% narrowing of the lumen of at least one of the major coronary arteries. For non-CAD patients, the ratios of atherosclerosis lesion to artery lumen were less than 30%. Baseline Characteristics of the Control and CAD Patients. Quantitate analysis using ImagePro plus software. All values are expressed as means ± s.e.m. The difference was determined by two-sided Student's t-test. p value vs control group.

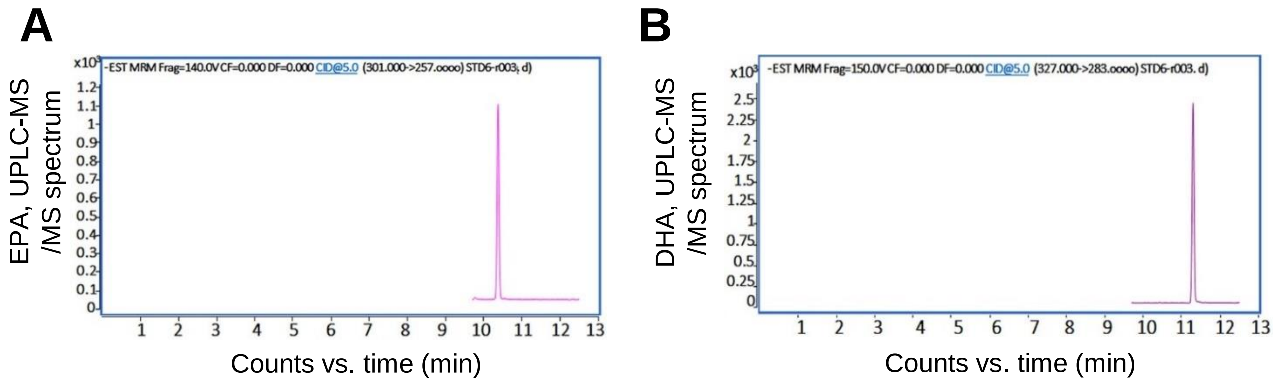

Supplementary Figure 1. Representative pictures showing EPA (**A**) and DHA (**B**) concentrations in serum according to gas chromatography.

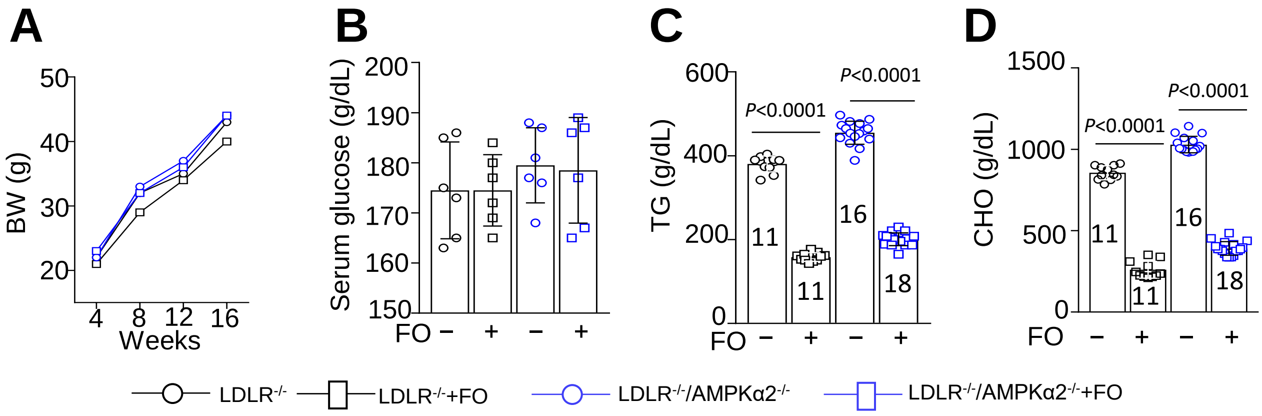

Supplementary Figure 2. Anti-atherosclerotic effect of FO is independent of serum lipid levels. Body weights(n=4) (**A**) and serum glucose(n=6) (**B**), CHO (**C**), and TG (**D**) levels were measured in LDLR$^{-/-}$ and LDLR$^{-/-}$/AMPKα2$^{-/-}$ mice fed a WD with or without 5% FO treatment. *p* value by two-sided Student's *t* test. Data are presented as the mean ± s.e.m. **CD**: Chow diet; **CHO**: total cholesterol; **FO**: fish oils; **LD**: lean diet; **PI-PC**: polyinosinic-polycytidylic acid; **TG**: triglycerides; **WD**: Western diet.

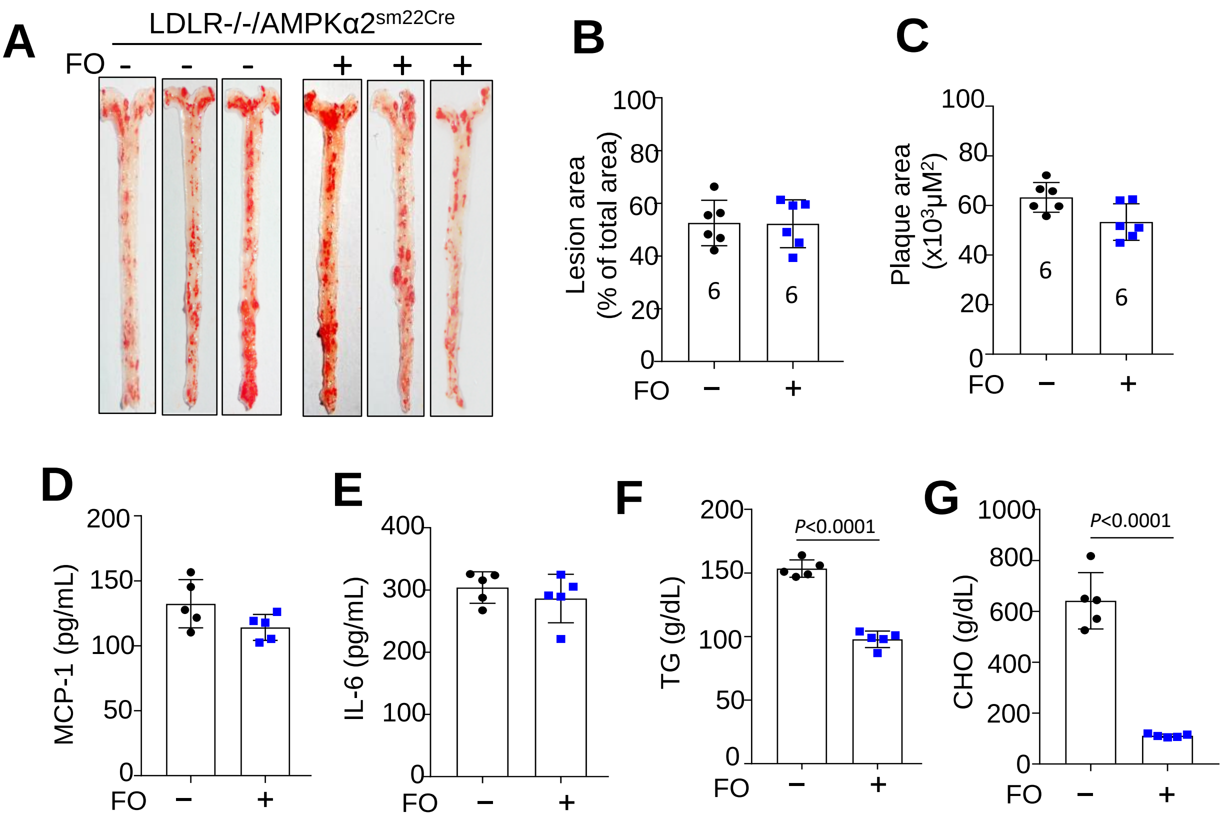

Supplementary Figure 3: AMPKα2 was required for the anti-atherosclerotic effects of FO in LDLR$^{-/-}$/AMPKα2^{sm22Cre} mice. (**A**) Representative images of Oil Red O staining of whole aortic tissue from LDLR$^{-/-}$/AMPKα2^{sm22Cre} with or without FO treatment (n = 6). (**B**) Quantification of aortic-lesion areas using Image-Pro plus software and between mice with and without FO treatment. (**C**) Percentages of lesion areas (ratio to lumen areas) of aortic roots were calculated for the two groups (n=6/group). Serum MCP-1 (**D**), IL-6 (**E**), TG (**F**)and CHO(**G**) levels were measured in LDLR$^{-/-}$/AMPKα2^{sm22Cre} mice fed a western diet with or without 5% FO treatment. All values are expressed as means ± s.e.m. *p* value by two-sided Student's *t* test.

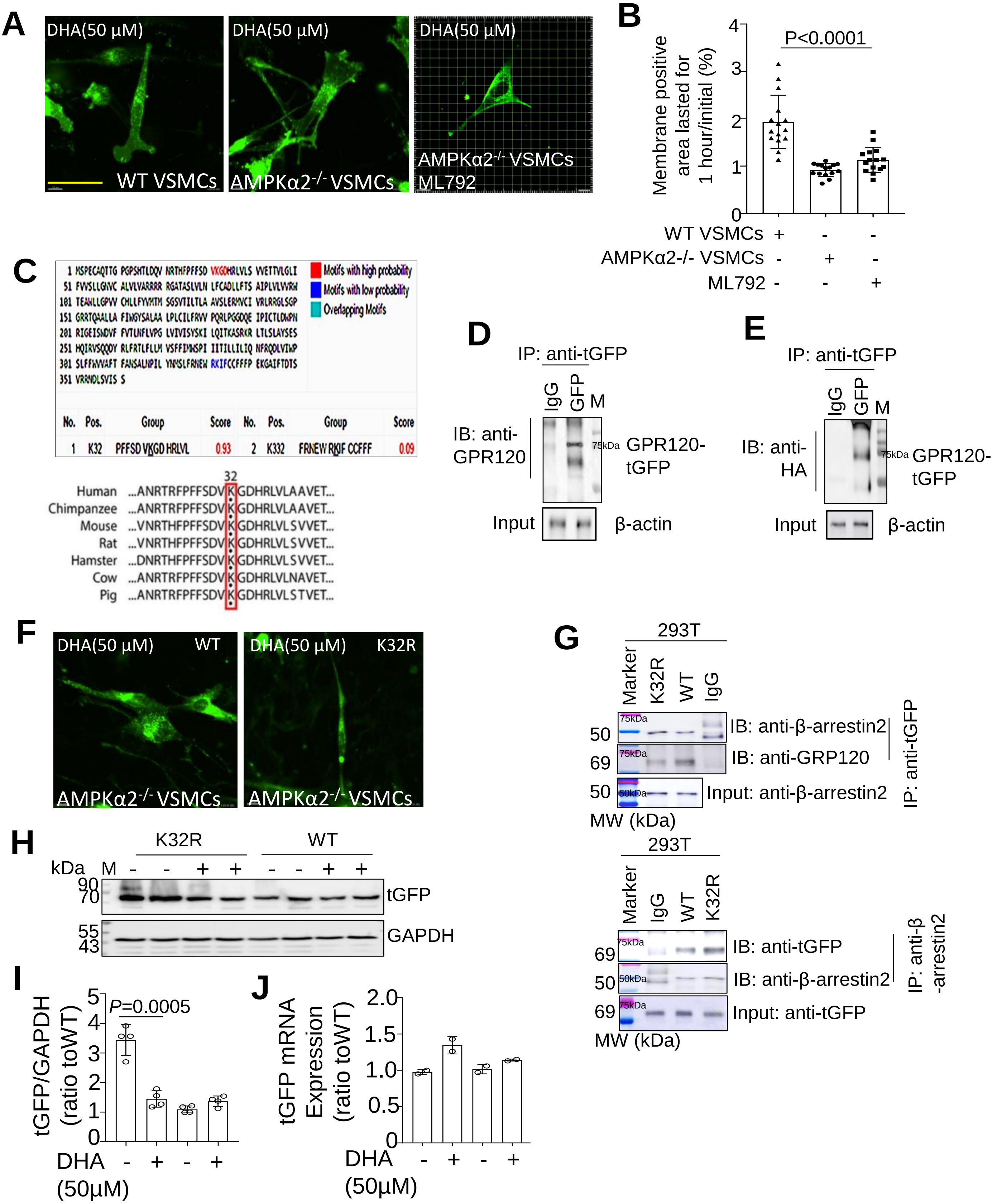
A
DHA(50 μM)
WT VSMCs
AMPKα2-/- VSMCs
AMPKα2-/- VSMCs ML792
B
Membrane positive area lasted for 1 hour/initial (%)
P<0.0001
WT VSMCs + - -
AMPKα2-/- VSMCs - + -
ML792 - - +
C
Motifs with high probability
Motifs with low probability
Overlapping Motifs
No. Pos. Group Score No. Pos. Group Score
1 K32 PFFSD VKGD HRLVL 0.93 2 K332 FRNEW RKIF CCFFF 0.09
32
Human ...ANRTRFPFFSDVKGDHRLVLAAVET...
Chimpanzee ...ANRTRFPFFSDVKGDHRLVLAAVET...
Mouse ...VNRTHFPFFSDVKGDHRLVLSVVET...
Rat ...VNRTHFPFFSDVKGDHRLVLSVVET...
Hamster ...DNRTHFPFFSDVKGDHRLVLSVVET...
Cow ...ANRTRFPFFSDVKGDHRLVLNAVET...
Pig ...ANRTRFPFFSDVKGDHRLVLSTVET...
D
IP: anti-tGFP
IgG GFP M
IB: anti-GPR120
75kDa GPR120-tGFP
Input β-actin
E
IP: anti-tGFP
IgG GFP M
IB: anti-HA
75kDa GPR120-tGFP
Input β-actin
F
DHA(50 μM) WT
DHA(50 μM) K32R
AMPKα2-/- VSMCs
G
293T
Marker K32R WT IgG
IB: anti-β-arrestin2
IB: anti-GRP120
Input: anti-β-arrestin2
IP: anti-tGFP
MW (kDa)
293T
Marker IgG WT K32R
IB: anti-tGFP
IB: anti-β-arrestin2
Input: anti-tGFP
IP: anti-β-arrestin2
MW (kDa)
H
K32R WT
kDa M - - + + - - + +
tGFP
GAPDH
I
tGFP/GAPDH (ratio toWT)
P=0.0005
DHA (50μM) - + - +
J
tGFP mRNA Expression (ratio toWT)
DHA (50μM) - + - +

Supplementary Figure 4. SUMOylation of GPR120 alters its localization and function in VSMCs. (**A**) A representative video picture showed DHA induced the translocalization of GPR120-tGFP plasmid transfected into WT VSMCs or AMPKα$2^{-/-}$ VSMCs, with or without ML792(1μM) . (**B**) Quantification of Membrane positive area lasted for 1 hour/initial in cells (n=15). (**C**) The Bioinformatics for probability analysis of SUMOylation sites in human GPR120 protein amino acid sequence. The putative SUMOylation site of GPR120, which is conserved among seven mammalian species. (**D, E**) IP assays for whole cell extracts from 293T cells co-transfected with GPR120-tGFP and SUMO2-HA vectors, showing SUMOylation of GPR120-tGFP. (**F**) A representative video picture showed that K32R/GPR120-tGFP or WT/GPR120-tGFP plasmid was transfected into the AMPKα$2^{-/-}$ VSMCs, and DHA-induced the translocalization of GPR120-tGFP fusion protein was investigated in AMPKα$2^{-/-}$ VSMCs. (**G**) IP assays for the interaction between GPR120 and β-arrestin 2 in 293T cells co-transfected K32R/GPR120-tGFP or WT/GPR120-tGFP with SUMO2-HA plasmids. (**H**) Western blotting (**I**) and quantification of tGFP in total fractions from AMPKα$2^{-/-}$ VSMCs when transfected by WT/GPR120-tGFP or K32R/GPR120-tGFP plasmid for 24hrs with or without DHA (50μM) treatment (n=4). All data shown are from one of at least two separate experiments.(**J**) Real-time PCR-based detection of tGFP mRNA in AMPK α $2^{-/-}$ VSMCs when transfected by WT/GPR120-tGFP or K32R/GPR120-tGFP plasmid for 24hrs (n=2). IP probe with either Anti-tGFP antibody or anti-β-arrestin2 antibody. IB blot with anti-GPR120, anti-tGFP or anti-β-arrestin2 respectively. **IB**: immunoblot; **IP**: immunoprecipitation; **WT**: wild type; **K32R**: lysine(K) 32 was mutated to arginine (R). **DHA**: docosahexaenoic acid.

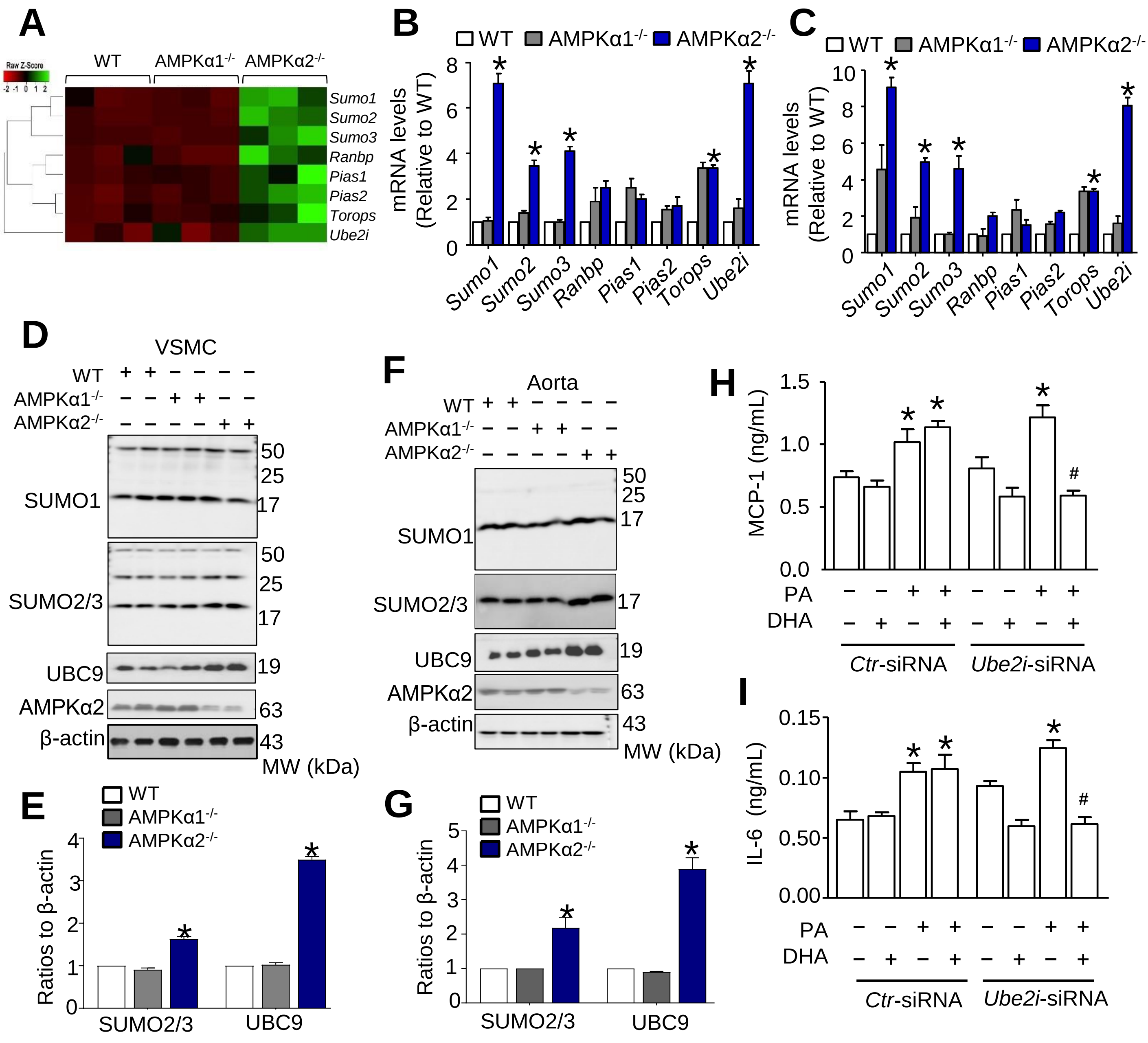

Supplemental Figure 5. **AMPKα2 deficiency increases the expression of UBC9 and SUMO2 in VSMCs and in aortic tissue.** (**A**) An mRNA microarray was used to detect gene-expression levels in WT, AMPKα1$^{-/-}$, and AMPKα2$^{-/-}$ VSMCs (*n* = 3 per group). Heat map showing correlative analysis of differential mRNA-expression levels for SUMOylation-related genes. Correlations were assessed using a nonparametric Spearman's test. mRNA levels of SUMOylation-related genes in VSMCs (**B**) or aortic tissues (**C**) from WT, AMPKα1$^{-/-}$, and AMPKα2$^{-/-}$ mice, determined by real-time PCR. (**D**-**G**)Western blotting and quantification of UBC9 and SUMO2/3 expression in VSMCs (**D**, **E**) and aortic tissues (**F**, **G**) of WT, AMPKα1$^{-/-}$, and AMPKα2$^{-/-}$ mice. ELISAs for MCP-1 (**H**) and IL-6 (**I**) secretion in AMPKα2$^{-/-}$ VSMCs transfected with *Ctr*-siRNA or *Ube2i*-siRNA, with or without PA (300 μM) and DHA (50 μM) treatment. All data shown represent one of at least two separate experiments. Quantification of western blot data was performed using Image-Pro plus software. Data are presented as the mean ± s.e.m. *$p < 0.05$ versus WT; #$p < 0.05$ versus PA VSMCs transfected with *Ube2i*-siRNA, by two-sided Student's t test. ***Ube2i*-siRNA**: siRNA against mouse Ube2i. All data shown are from one of at least two separate experiments.

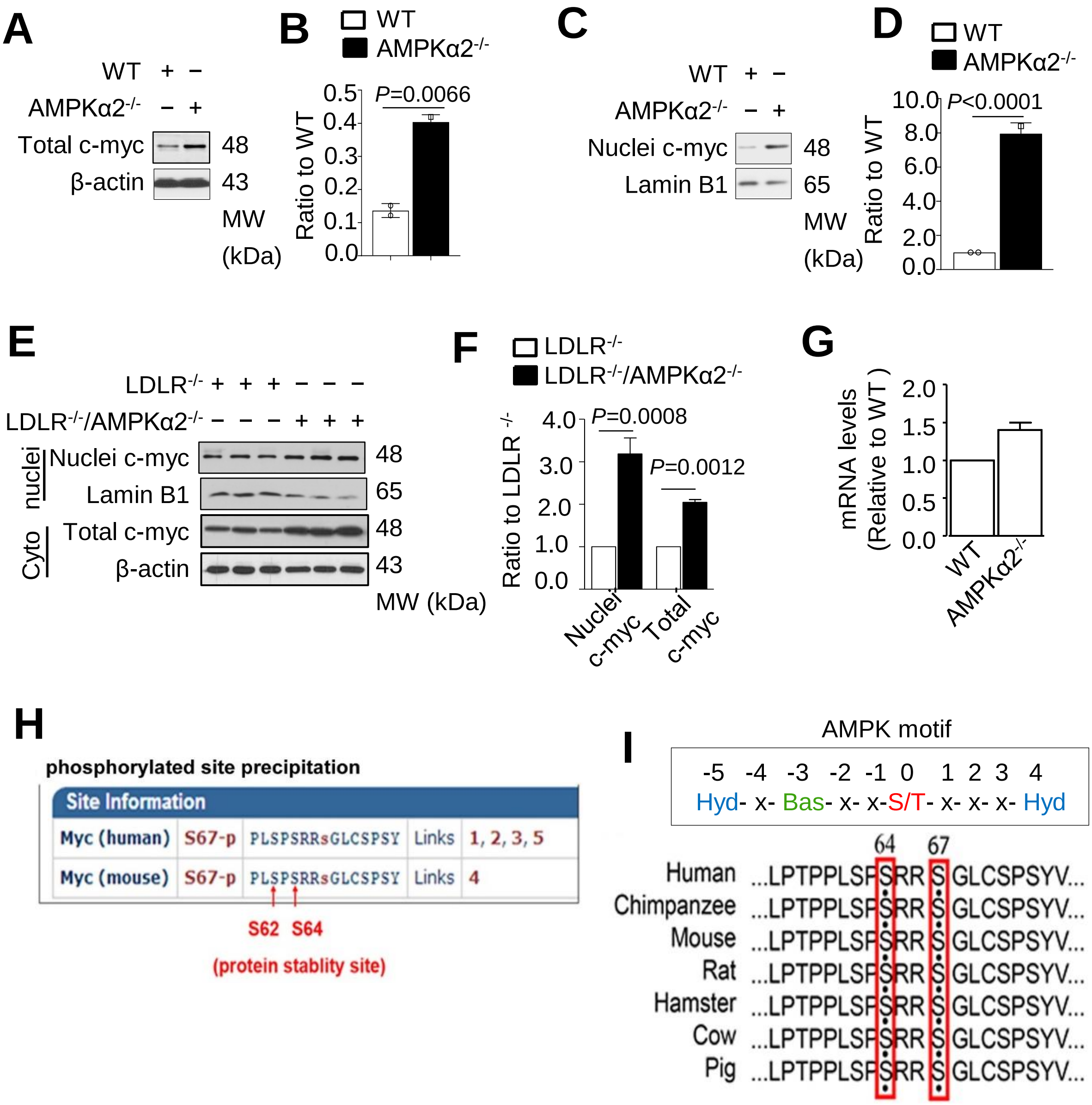

Supplementary Figure 6. C-myc S67 phosphorylation was essential for AMPKα2-controlled expression of UBC9 and SUMO2/3. Western blotting and quantification of c-myc (**A**) and nuclear pS62-c-myc (**B**) in total and nuclear fractions from WT and AMPKα2$^{-/-}$ VSMCs. (**C**) Western blotting and quantification of c-myc and nuclear pS62-c-myc in aortic tissues from LDLR$^{-/-}$ or LDLR$^{-/-}$/AMPKα2$^{-/-}$ mice. (**D**) Real-time PCR-based detection of *Myc* mRNA in WT and AMPKα2$^{-/-}$ VSMCs. (**E**) Western blotting (**F**) and quantification of of c-myc and nuclear pS62-c-myc in total and nuclear fractions from WT and AMPKα2$^{-/-}$ aortic artery. (**G**) mRNA expression of *c-Myc* in WT and AMPKα2$^{-/-}$ VSMCs. (**H**) Bioinformatics analysis of human and mouse c-myc amino acid sequences to search for putative phosphorylation sites targeted by AMPKα. (**I**) Serine at amino acids 64 or 67 in the c-myc protein are conserved among seven mammalian species. All data shown represent one of at least three separate experiments. Quantification of western blots was performed using Image-Pro plus software. Data are presented as the mean ± s.e.m. *p* value by two-sided Student's *t* tests. **AICAR**: 5-aminoimidazole-4-carboxamide 1-β-D-ribofuranoside; ***Myc*-siRN**A: siRNA against mouse *Myc.*

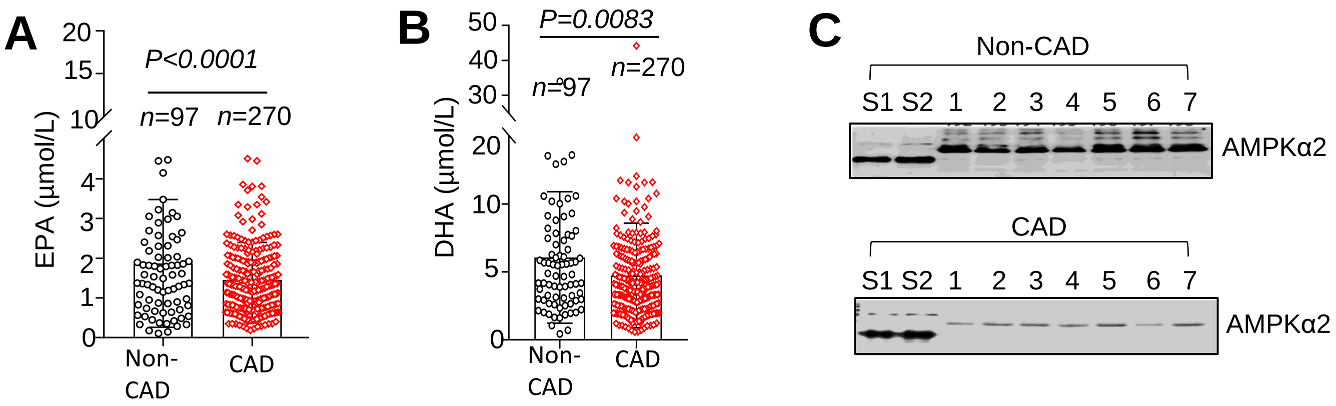

Supplementarry Figure 7. the level of AMPKα2 determined the FO effects on CAD. Plasma EPA (**A**) and DHA (**B**) concentrations were measured by gas chromatography in Non-CAD and CAD patients. AMPKα2 expression(**C**) was detected by western blotting in platelet protein from rich platelet plasma in Non-CAD and CAD patients. The boxes denote the minimum, median, and maximum values, and error bars represent the standard error of the mean (s.e.m.); *p* value by a two-sided Student's *t* test. S1 means AMPKα2 standard protein 50 pg/mL, S2 represents AMPKα2 standard protein 100 pg/mL.
