# Supplementary material for: AMPKα2 controls the anti-atherosclerotic effects of fish oils by modulating the SUMOylation of GPR120
Source: Nat Commun. 2022 Dec 13;13:7721. doi: 10.1038/s41467-022-34996-x (PMC9747961; doi:10.1038/s41467-022-34996-x)
Supplement: Supplementary file 2 — Description of Additional Supplementary Files [file 41467_2022_34996_MOESM2_ESM.pdf]

Description of Additional Supplementary Files

File Name: Supplementary Movie 1
Description: **(in Supplementary Figure 4A)**: A representative video picture showed DHA induced the translocalization of GPR120-tGFP plasmid transfected into WT VSMCs.

File Name: Supplementary Movie 2
Description: **(in Supplementary Figure 4A)**: A representative video picture showed DHA induced the translocalization of GPR120-tGFP plasmid transfected into AMPKα$2^{-/-}$ VSMCs.

File Name: Supplementary Movie 3
Description: **(in Supplementary Figure 4A)**: A representative video picture showed DHA induced the translocalization of GPR120-tGFP plasmid transfected into AMPKα$2^{-/-}$ VSMCs, with ML792(1μM).

File Name: Supplementary Movie 4
Description: **(in Supplementary Figure 4F)**: A representative video picture showed that WT/GPR120-tGFP plasmid was transfected into the AMPKα$2^{-/-}$ VSMCs, and DHA-induced the translocalization of GPR120-tGFP fusion protein was investigated in AMPKα$2^{-/-}$ VSMCs.

File Name: Supplementary Movie 5
Description: **(in Supplementary Figure 4F)**: A representative video picture showed that K32R/GPR120-tGFP plasmid was transfected into the AMPKα$2^{-/-}$ VSMCs, and DHA-induced the translocalization of GPR120-tGFP fusion protein was investigated in AMPKα$2^{-/-}$ VSMCs.
