# Supplementary material for: AMPKα2 controls the anti-atherosclerotic effects of fish oils by modulating the SUMOylation of GPR120
Source: Nat Commun. 2022 Dec 13;13:7721. doi: 10.1038/s41467-022-34996-x (PMC9747961; doi:10.1038/s41467-022-34996-x)
Supplement: Supplementary file 8 — Reporting Summary [file 41467_2022_34996_MOESM8_ESM.pdf]

nature portfolio

Corresponding author(s): Yaling Han

Last updated by author(s): Aug 31, 2022

Reporting Summary

Nature Portfolio wishes to improve the reproducibility of the work that we publish. This form provides structure for consistency and transparency in reporting. For further information on Nature Portfolio policies, see our Editorial Policies and the Editorial Policy Checklist.

Statistics

For all statistical analyses, confirm that the following items are present in the figure legend, table legend, main text, or Methods section.

n/a	Confirmed	
☐	☒	The exact sample size (*n*) for each experimental group/condition, given as a discrete number and unit of measurement
☐	☒	A statement on whether measurements were taken from distinct samples or whether the same sample was measured repeatedly
☐	☒	The statistical test(s) used AND whether they are one- or two-sided<br>*Only common tests should be described solely by name; describe more complex techniques in the Methods section.*
☐	☒	A description of all covariates tested
☐	☒	A description of any assumptions or corrections, such as tests of normality and adjustment for multiple comparisons
☐	☒	A full description of the statistical parameters including central tendency (e.g. means) or other basic estimates (e.g. regression coefficient) AND variation (e.g. standard deviation) or associated estimates of uncertainty (e.g. confidence intervals)
☐	☒	For null hypothesis testing, the test statistic (e.g. *F*, *t*, *r*) with confidence intervals, effect sizes, degrees of freedom and *P* value noted<br>*Give P values as exact values whenever suitable.*
☒	☐	For Bayesian analysis, information on the choice of priors and Markov chain Monte Carlo settings
☒	☐	For hierarchical and complex designs, identification of the appropriate level for tests and full reporting of outcomes
☐	☒	Estimates of effect sizes (e.g. Cohen's *d*, Pearson's *r*), indicating how they were calculated

Our web collection on statistics for biologists contains articles on many of the points above.

Software and code

Policy information about availability of computer code

Data collection	GraphPad Prism 5 (GraphPad Software, Inc., La Jolla, CA) or R version 3.1.1 (http://www.rproject.org/).
Data analysis	Statistical analyses were performed with GraphPad Prism 5 (GraphPad Software, Inc., La Jolla, CA) or R version 3.1.1 (http://www.rproject.org/).

For manuscripts utilizing custom algorithms or software that are central to the research but not yet described in published literature, software must be made available to editors and reviewers. We strongly encourage code deposition in a community repository (e.g. GitHub). See the Nature Portfolio guidelines for submitting code & software for further information.

Data

Policy information about availability of data

All manuscripts must include a data availability statement. This statement should provide the following information, where applicable:

- Accession codes, unique identifiers, or web links for publicly available datasets
- A description of any restrictions on data availability
- For clinical datasets or third party data, please ensure that the statement adheres to our policy

Serum DHA and EPA were detected by Bio-tech Technical Company, Wuhan, China. Uncropped versions of all gels and blots are provided in Source data. All other data are available from the corresponding authors upon reasonable request.

Human research participants

Policy information about studies involving human research participants and Sex and Gender in Research.

Reporting on sex and gender	A total of 349 patients with 270 CAD and 79 Non-CAD individuals, aged between 29 and 75 years, were enrolled in the study from April 2017 to June 2019. The patients were unrelated Han individuals from the General Hospital of China. Female numbers is 38.7% in non-CAD and is 22.2% in CAD group.
Population characteristics	All the subjects had undergone coronary angiography for the evaluation of suspected or established CAD at Northern Hospital, Shenyang, China. Detailed sample-by-sample information on all patients and key clinicopathologic characteristics of each cohort are also summarised in Supplementary Table S2.
Recruitment	Subjects who had inflammatory diseases, valvular heart disease, cancers, or rheumatoid arthritis were excluded. The inclusion and exclusion criteria are listed in Supplementary Table 1.
Ethics oversight	The study was approved by the Regional Committee for Medical Research Ethics in the Northeast Region Hospital of China(K2017-16). Informed consents were obtained from all participants in the study.The study was authorized, and the study design and conduct complied with all relevant regulations regarding the use of human study participants and was conducted in accordance with the criteria set by the Declaration of Helsinki.

Note that full information on the approval of the study protocol must also be provided in the manuscript.

Field-specific reporting

Please select the one below that is the best fit for your research. If you are not sure, read the appropriate sections before making your selection.

☒ Life sciences ☐ Behavioural & social sciences ☐ Ecological, evolutionary & environmental sciences

For a reference copy of the document with all sections, see nature.com/documents/nr-reporting-summary-flat.pdf

Life sciences study design

All studies must disclose on these points even when the disclosure is negative.

Sample size	A total of 349 patients with 270 CAD and 79 Non-CAD individuals, aged between 29 and 75 years, were enrolled in the study from April 2017 to June 2019 based on the collected baseline factors. Low-density lipoprotein receptor knockout (LDLR−/−) (C57BL/6 background) mice (5-6 weeks of age) were purchased from the Jackson Laboratory (Bar Harbor, ME). AMPKa1−/− and AMPKa2−/− mice were generated and backcrossed to a C57BL/6 background for at least ten generations.
Data exclusions	All patients had undergone coronary angiography for the evaluation of suspected or established CAD at Northern Hospital, Shenyang, China. Subjects who had inflammatory diseases, valvular heart disease, cancers, or rheumatoid arthritis were excluded.
Replication	All data shown represent one of at least two separate experiments.
Randomization	A total of 349 patients with 270 CAD and 79 Non-CAD individuals, aged between 29 and 75 years, were enrolled in the study from April 2017 to June 2019. Complete clinical histories were obtained from all subjects, the baseline characteristics are shown in Supplemental Table 2.
Blinding	The investigators were blinded to group allocation during data collection and analysis.

Reporting for specific materials, systems and methods

We require information from authors about some types of materials, experimental systems and methods used in many studies. Here, indicate whether each material, system or method listed is relevant to your study. If you are not sure if a list item applies to your research, read the appropriate section before selecting a response.

Materials & experimental systems

n/a	Involved in the study	
☐	☒	Antibodies
☐	☒	Eukaryotic cell lines
☐	☐	Palaeontology and archaeology
☐	☒	Animals and other organisms
☒	☐	Clinical data
☐	☐	Dual use research of concern

Methods

n/a	Involved in the study	
☐	☐	ChIP-seq
☐	☐	Flow cytometry
☐	☐	MRI-based neuroimaging

Antibodies

Antibodies used

Anti-tGFP (TA150039) was purchased from OriGene (Rockville, MD). Anti-AMPK□ (#5831), anti-pAMPK□-Thr172 (#2535), anti-AMPK□1 (#2795), anti-AMPK□2 (#2757), anti-c-myc (#5605), anti-pc-myc-Ser62 (#13748), anti-UBC9 (#4786), anti-IL-6 (#13797), anti-NaK ATPase (#7074), anti-□-smooth muscle actin (SMA; #56856), anti-SUMO2/3 (# ?) and anti-MCP-1 (#12838) were purchased from Cell Signaling Technology Inc. (Danvers, MA). Anti-β-arrestin 2 (C16D9) rabbit mAb (Cell Signaling Technology) for the western blotting analysis, and sc-365445 (Santa Cruz Biotechnology) for the IP assay. Anti-β-arrestin 1 (D8O3J) rabbit mAb (Cell Signaling Technology). Anti-□-actin (sc-47778), and anti-SUMO1 (sc-5380) were purchased from Santa Cruz Biotechnology, Inc. (Dallas, TX). anti-GPR120 (sc-390752), Anti-SUMO2/3 immunoprecipitation (IP) beads (#BK-162) were purchased from Cellskeleton, Inc and antibody (sc-50331) were purchased from Santa Cruz Biotechnology, Inc. GW9508 (G9797) and AICAR (A9978) were purchased from Sigma-Aldrich (St. Louis, MO). DHA (CAS6217-54-5) was purchased from Cayman Chemical Company (Ann Arbor, MI). Anti-CD68 (14-0681-80) were purchased from Affymetrix-ebioscience.

Validation

The validation of each primary antibody was done in the manuscript in mice tissue or cell lines in Figure 2-Figure 7, Supplemental Figure3-supplemental Figue 7.

Eukaryotic cell lines

Policy information about cell lines and Sex and Gender in Research

Cell line source(s)

Mouse primary VSMCs were isolated from cultured explants of aortas from 8- to 10-week-old wild-type C57BL/6J (WT), AMPKa1−/−, and AMPKa2−/− mice.

Authentication

The authentication were done by detecting the expression of VSMC marker protein and AMPKa1 or AMPKa2 protein

Mycoplasma contamination

negative

Commonly misidentified lines
(See ICLAC register)

no

Palaeontology and Archaeology

Specimen provenance

Provide provenance information for specimens and describe permits that were obtained for the work (including the name of the issuing authority, the date of issue, and any identifying information). Permits should encompass collection and, where applicable, export.

Specimen deposition

Indicate where the specimens have been deposited to permit free access by other researchers.

Dating methods

If new dates are provided, describe how they were obtained (e.g. collection, storage, sample pretreatment and measurement), where they were obtained (i.e. lab name), the calibration program and the protocol for quality assurance OR state that no new dates are provided.

☐ Tick this box to confirm that the raw and calibrated dates are available in the paper or in Supplementary Information.

Ethics oversight

Identify the organization(s) that approved or provided guidance on the study protocol, OR state that no ethical approval or guidance was required and explain why not.

Note that full information on the approval of the study protocol must also be provided in the manuscript.

Animals and other research organisms

Policy information about studies involving animals; ARRIVE guidelines recommended for reporting animal research, and Sex and Gender in Research

Laboratory animals

Low-density lipoprotein receptor knockout (LDLR−/−) (C57BL/6 background) mice (5-6 weeks of age) were purchased from the

Laboratory animals	Jackson Laboratory (Bar Harbor, ME). Prkaa2 floxed (AMPKα2fl/fl) mice, LDLR−/−/ AMPKα2−/− mice and LDLR−/−/AMPKα2−/−sm22Cre (VSMC-specific Prkaa2 knockout) mice were kindly provided by Dr. Zou 's lab and backcrossed to a C57BL/6 background for at least ten generations.
Wild animals	No wild animals were used.
Reporting on sex	All male mice were fed a western diet (contains 0.21% cholesterol) for 12 weeks to induce atherosclerosis and grouped with or without 5% FO administration.
Field-collected samples	Mice were housed in a specific-pathogen-free facility on a 12-h light/dark cycle and allowed to acclimate for 1-2 weeks.
Ethics oversight	The animal protocol was reviewed and approved by General Hospital of Shenyang Northern Theater Command Institute Animal Care and Use Committee.

Note that full information on the approval of the study protocol must also be provided in the manuscript.

Dual use research of concern

Policy information about dual use research of concern

Hazards

Could the accidental, deliberate or reckless misuse of agents or technologies generated in the work, or the application of information presented in the manuscript, pose a threat to:

No	Yes	
☒	☐	Public health
☒	☐	National security
☒	☐	Crops and/or livestock
☒	☐	Ecosystems
☒	☐	Any other significant area

Experiments of concern

Does the work involve any of these experiments of concern:

No	Yes	
☒	☐	Demonstrate how to render a vaccine ineffective
☒	☐	Confer resistance to therapeutically useful antibiotics or antiviral agents
☒	☐	Enhance the virulence of a pathogen or render a nonpathogen virulent
☒	☐	Increase transmissibility of a pathogen
☒	☐	Alter the host range of a pathogen
☒	☐	Enable evasion of diagnostic/detection modalities
☒	☐	Enable the weaponization of a biological agent or toxin
☒	☐	Any other potentially harmful combination of experiments and agents

ChIP-seq

Data deposition

☐ Confirm that both raw and final processed data have been deposited in a public database such as GEO.

☐ Confirm that you have deposited or provided access to graph files (e.g. BED files) for the called peaks.

Data access links<br>*May remain private before publication.*	*For "Initial submission" or "Revised version" documents, provide reviewer access links. For your "Final submission" document, provide a link to the deposited data.*
Files in database submission	*Provide a list of all files available in the database submission.*
Genome browser session<br>(e.g. UCSC)	*Provide a link to an anonymized genome browser session for "Initial submission" and "Revised version" documents only, to enable peer review. Write "no longer applicable" for "Final submission" documents.*

Methodology

Replicates	*Describe the experimental replicates, specifying number, type and replicate agreement.*

Sequencing depth | *Describe the sequencing depth for each experiment, providing the total number of reads, uniquely mapped reads, length of reads and whether they were paired- or single-end.*

Antibodies | *Describe the antibodies used for the ChIP-seq experiments; as applicable, provide supplier name, catalog number, clone name, and lot number.*

Peak calling parameters | *Specify the command line program and parameters used for read mapping and peak calling, including the ChIP, control and index files used.*

Data quality | *Describe the methods used to ensure data quality in full detail, including how many peaks are at FDR 5% and above 5-fold enrichment.*

Software | *Describe the software used to collect and analyze the ChIP-seq data. For custom code that has been deposited into a community repository, provide accession details.*

Flow Cytometry

Plots

Confirm that:

- [ ] The axis labels state the marker and fluorochrome used (e.g. CD4-FITC).
- [ ] The axis scales are clearly visible. Include numbers along axes only for bottom left plot of group (a 'group' is an analysis of identical markers).
- [ ] All plots are contour plots with outliers or pseudocolor plots.
- [ ] A numerical value for number of cells or percentage (with statistics) is provided.

Methodology

Sample preparation | *Describe the sample preparation, detailing the biological source of the cells and any tissue processing steps used.*

Instrument | *Identify the instrument used for data collection, specifying make and model number.*

Software | *Describe the software used to collect and analyze the flow cytometry data. For custom code that has been deposited into a community repository, provide accession details.*

Cell population abundance | *Describe the abundance of the relevant cell populations within post-sort fractions, providing details on the purity of the samples and how it was determined.*

Gating strategy | *Describe the gating strategy used for all relevant experiments, specifying the preliminary FSC/SSC gates of the starting cell population, indicating where boundaries between "positive" and "negative" staining cell populations are defined.*

- [ ] Tick this box to confirm that a figure exemplifying the gating strategy is provided in the Supplementary Information.

Magnetic resonance imaging

Experimental design

Design type | *Indicate task or resting state; event-related or block design.*

Design specifications | *Specify the number of blocks, trials or experimental units per session and/or subject, and specify the length of each trial or block (if trials are blocked) and interval between trials.*

Behavioral performance measures | *State number and/or type of variables recorded (e.g. correct button press, response time) and what statistics were used to establish that the subjects were performing the task as expected (e.g. mean, range, and/or standard deviation across subjects).*

Acquisition

Imaging type(s) | *Specify: functional, structural, diffusion, perfusion.*

Field strength | *Specify in Tesla*

Sequence & imaging parameters | *Specify the pulse sequence type (gradient echo, spin echo, etc.), imaging type (EPI, spiral, etc.), field of view, matrix size, slice thickness, orientation and TE/TR/flip angle.*

Area of acquisition | *State whether a whole brain scan was used OR define the area of acquisition, describing how the region was determined.*

Diffusion MRI ☐ Used ☐ Not used

Preprocessing

Preprocessing software	*Provide detail on software version and revision number and on specific parameters (model/functions, brain extraction, segmentation, smoothing kernel size, etc.).*
Normalization	*If data were normalized/standardized, describe the approach(es): specify linear or non-linear and define image types used for transformation OR indicate that data were not normalized and explain rationale for lack of normalization.*
Normalization template	*Describe the template used for normalization/transformation, specifying subject space or group standardized space (e.g. original Talairach, MNI305, ICBM152) OR indicate that the data were not normalized.*
Noise and artifact removal	*Describe your procedure(s) for artifact and structured noise removal, specifying motion parameters, tissue signals and physiological signals (heart rate, respiration).*
Volume censoring	*Define your software and/or method and criteria for volume censoring, and state the extent of such censoring.*

Statistical modeling & inference

Model type and settings	*Specify type (mass univariate, multivariate, RSA, predictive, etc.) and describe essential details of the model at the first and second levels (e.g. fixed, random or mixed effects; drift or auto-correlation).*
Effect(s) tested	*Define precise effect in terms of the task or stimulus conditions instead of psychological concepts and indicate whether ANOVA or factorial designs were used.*

Specify type of analysis: ☐ Whole brain ☐ ROI-based ☐ Both

Statistic type for inference (See Eklund et al. 2016)	*Specify voxel-wise or cluster-wise and report all relevant parameters for cluster-wise methods.*
Correction	*Describe the type of correction and how it is obtained for multiple comparisons (e.g. FWE, FDR, permutation or Monte Carlo).*

Models & analysis

n/a	Involved in the study
☐	☐ Functional and/or effective connectivity
☐	☐ Graph analysis
☐	☐ Multivariate modeling or predictive analysis

Functional and/or effective connectivity	*Report the measures of dependence used and the model details (e.g. Pearson correlation, partial correlation, mutual information).*
Graph analysis	*Report the dependent variable and connectivity measure, specifying weighted graph or binarized graph, subject- or group-level, and the global and/or node summaries used (e.g. clustering coefficient, efficiency, etc.).*
Multivariate modeling and predictive analysis	*Specify independent variables, features extraction and dimension reduction, model, training and evaluation metrics.*
