# Supplementary material for: AMPKα2 controls the anti-atherosclerotic effects of fish oils by modulating the SUMOylation of GPR120
Source: Nat Commun. 2022 Dec 13;13:7721. doi: 10.1038/s41467-022-34996-x (PMC9747961; doi:10.1038/s41467-022-34996-x)
Supplement: Supplementary file 9 — Source Data [file 41467_2022_34996_MOESM9_ESM.zip › source data file/Original data for figures and supplemental.pdf]

Figure 1A

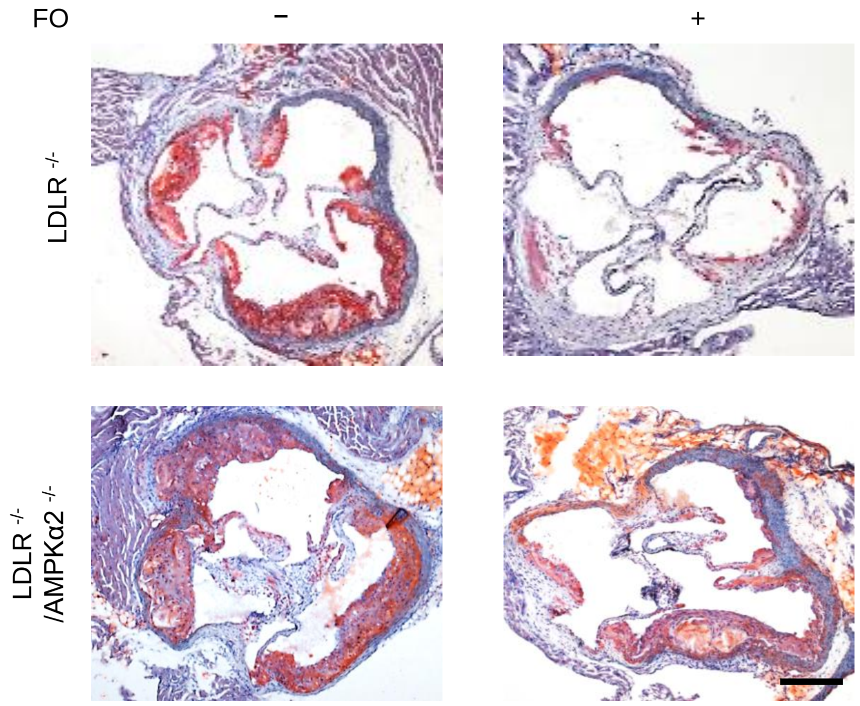

Figure 1E

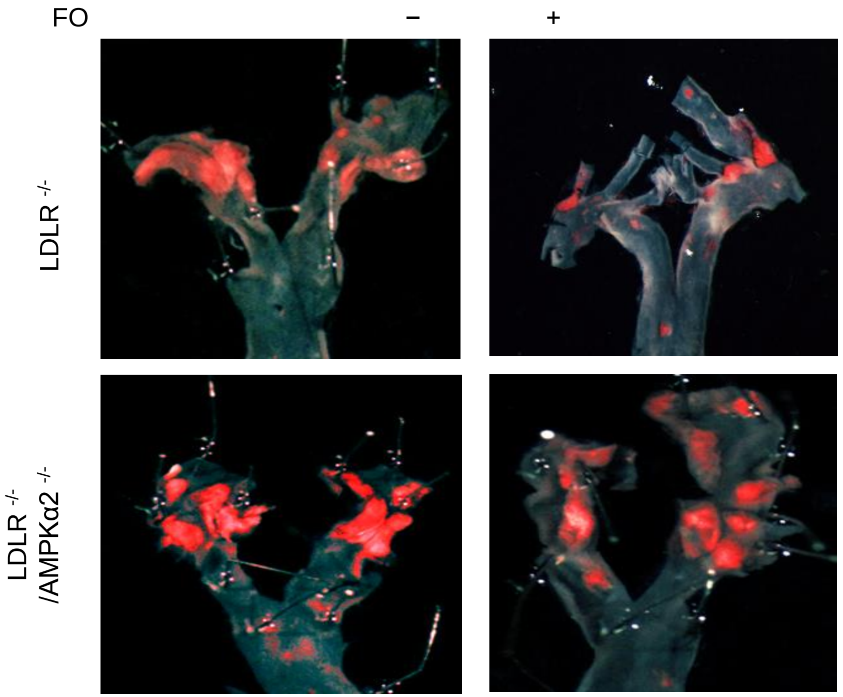

Figure 1I

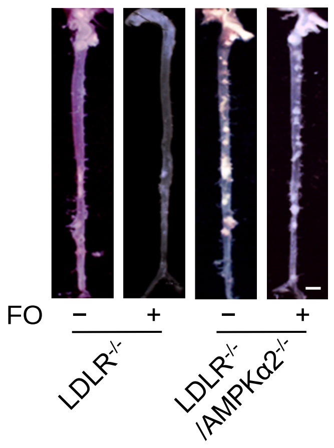

Figure 2C

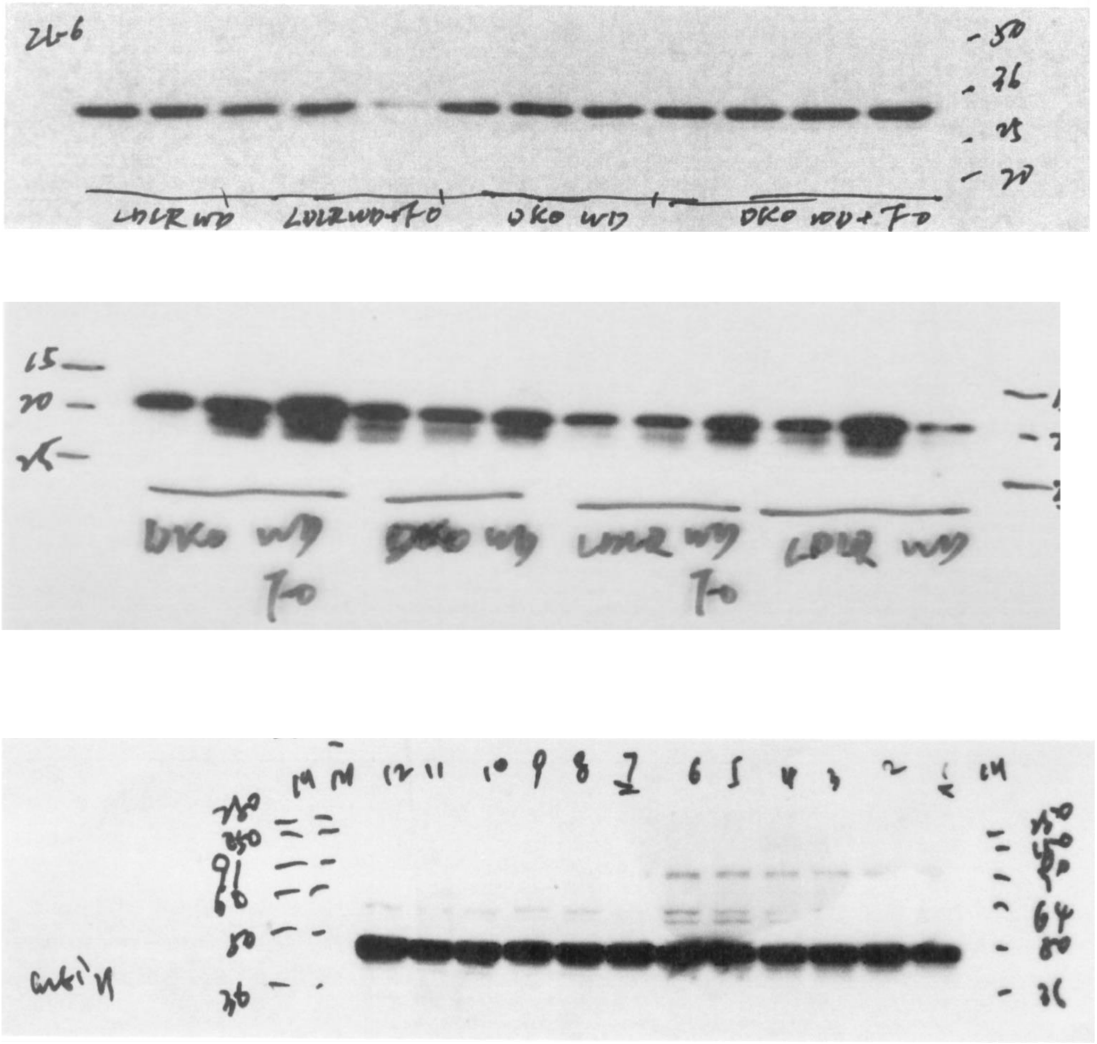

IL-6

MCP1

actin

Figure2H

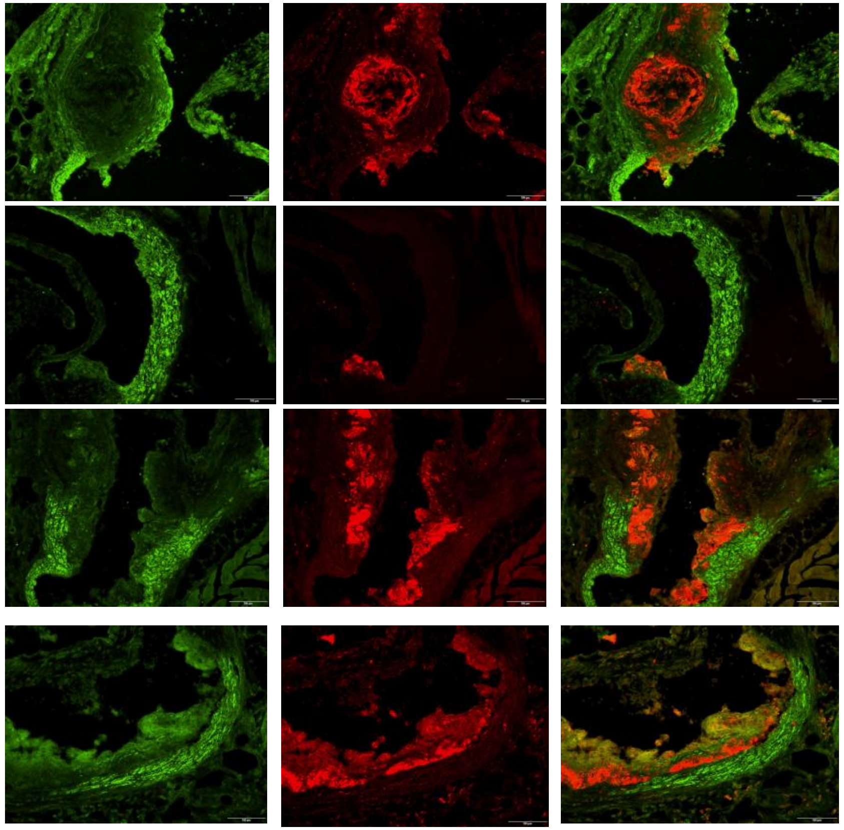

Figure 3A

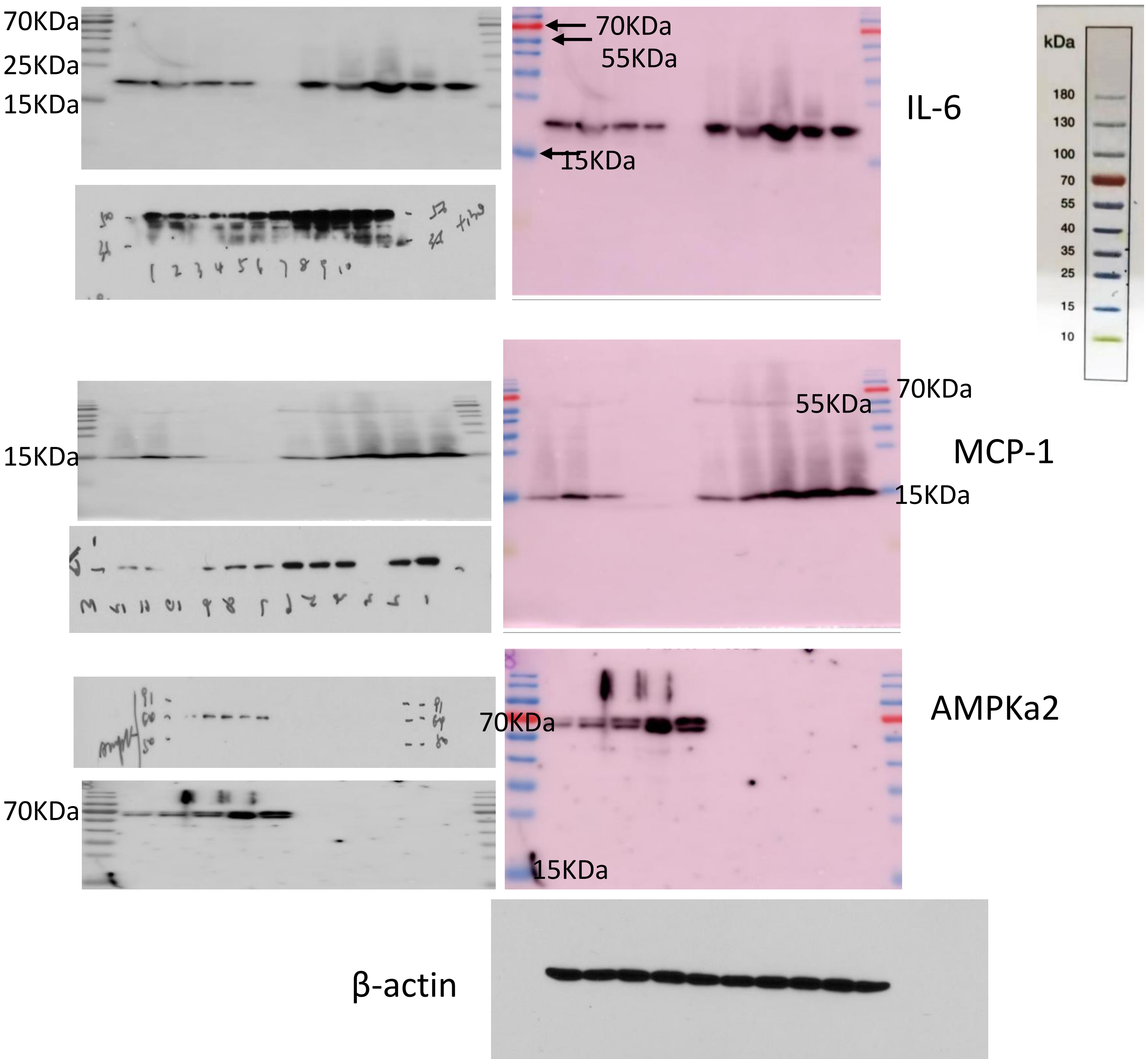

70KDa
25KDa
15KDa
70KDa
55KDa
15KDa
IL-6
kDa
180
130
100
70
55
40
35
25
15
10
15KDa
70KDa
55KDa
MCP-1
15KDa
70KDa
AMPKa2
70KDa
15KDa
β-actin

Figure 4B

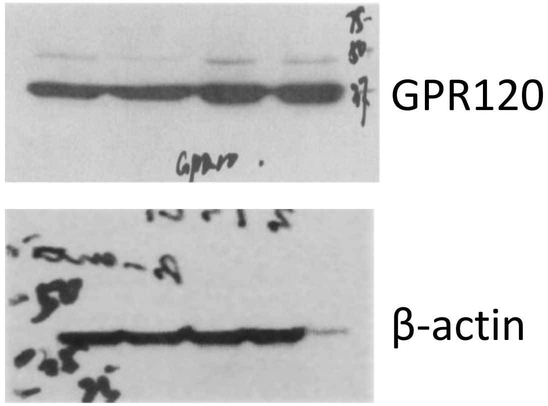

Figure 4D

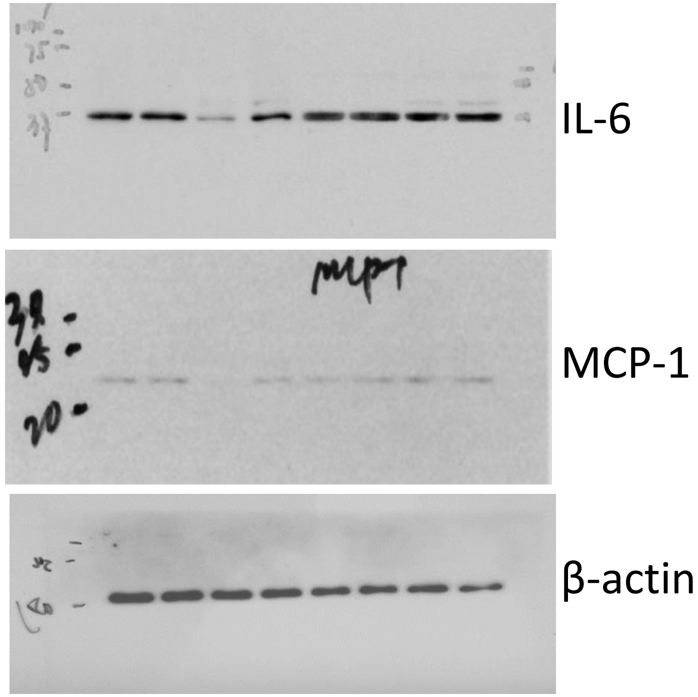

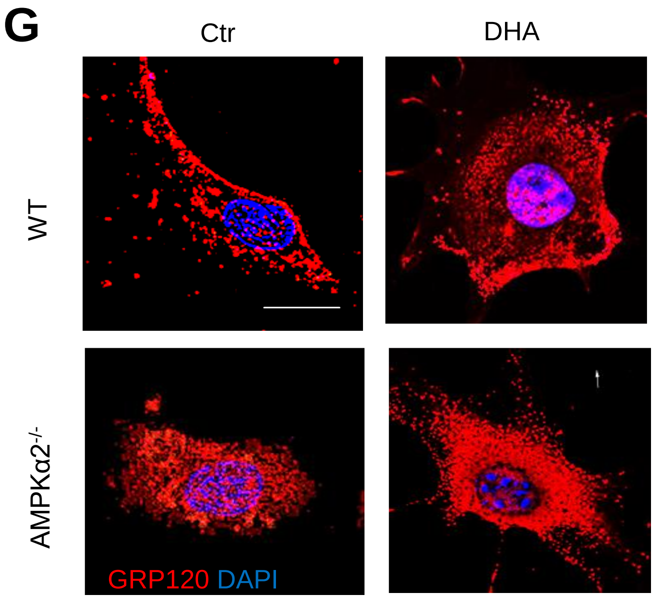

Figure 4H

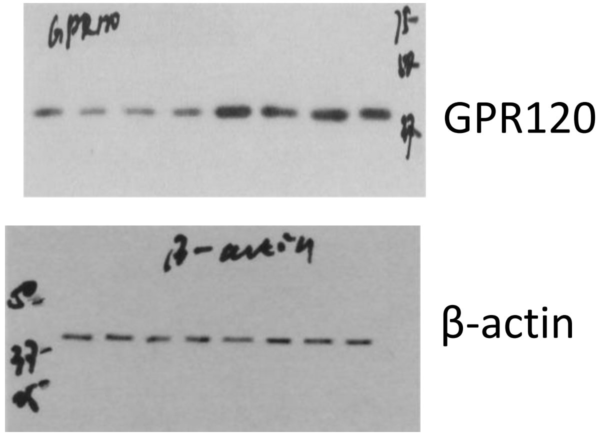

Figure 4J

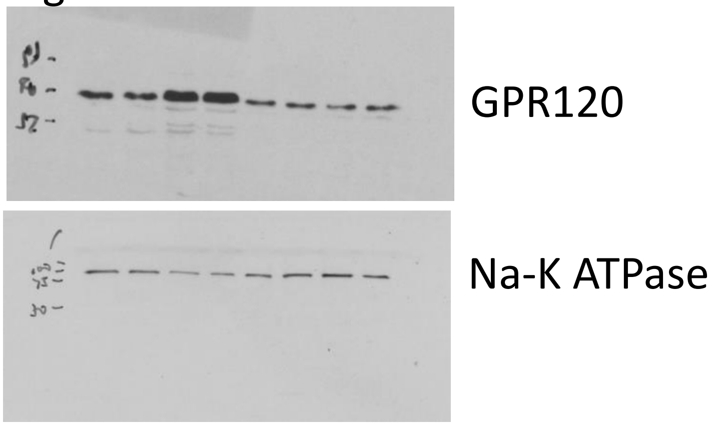

Figure

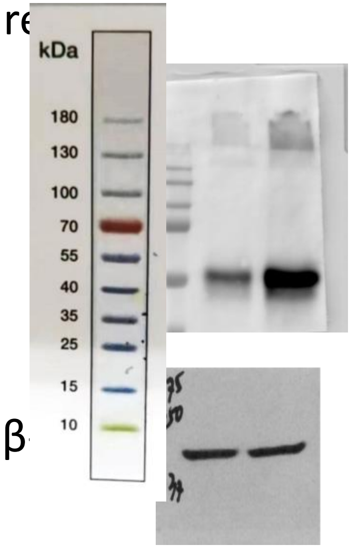

β-arrestin

β

Figure 4N

IgG wt AMPKa2-/-

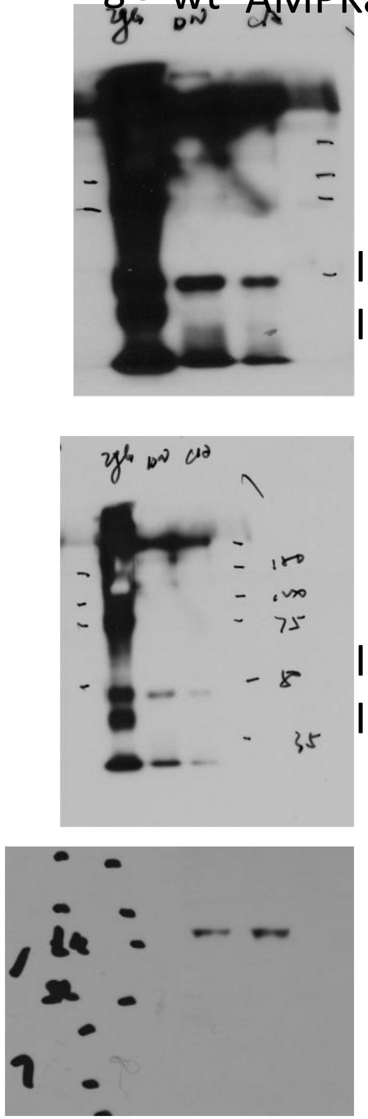

IP: tGFP
IB: tGFP

IP: tGFP
IB: β-arrestin

Input: β-actin

Figure 4O

IgG wt AMPKa2-/-

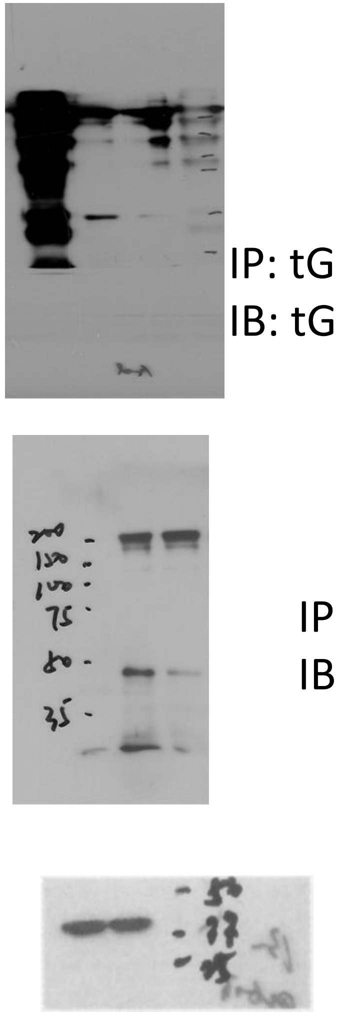

IP: tGFP
IB: tGFP

IP: tGFP
IB: β-arrestin

Input: β-actin

Figure 5A and 5B

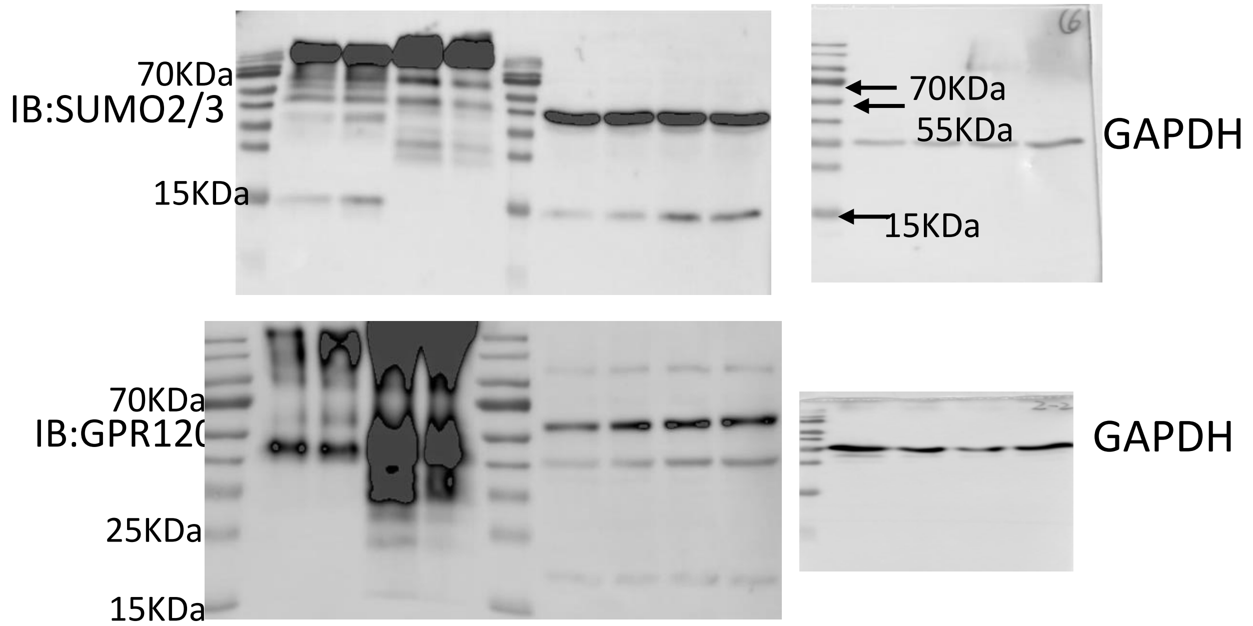

Figure 5C

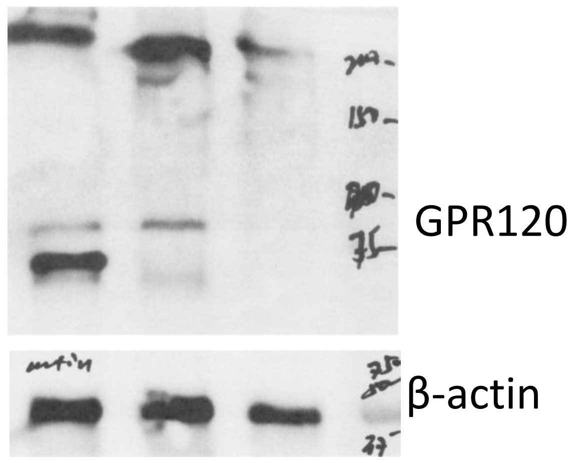

Figure 5D

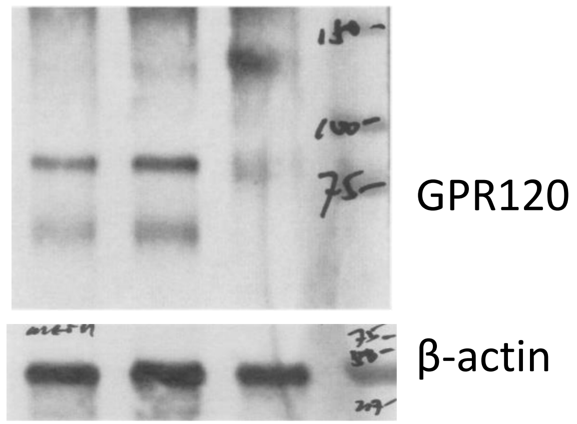

Figure 5E

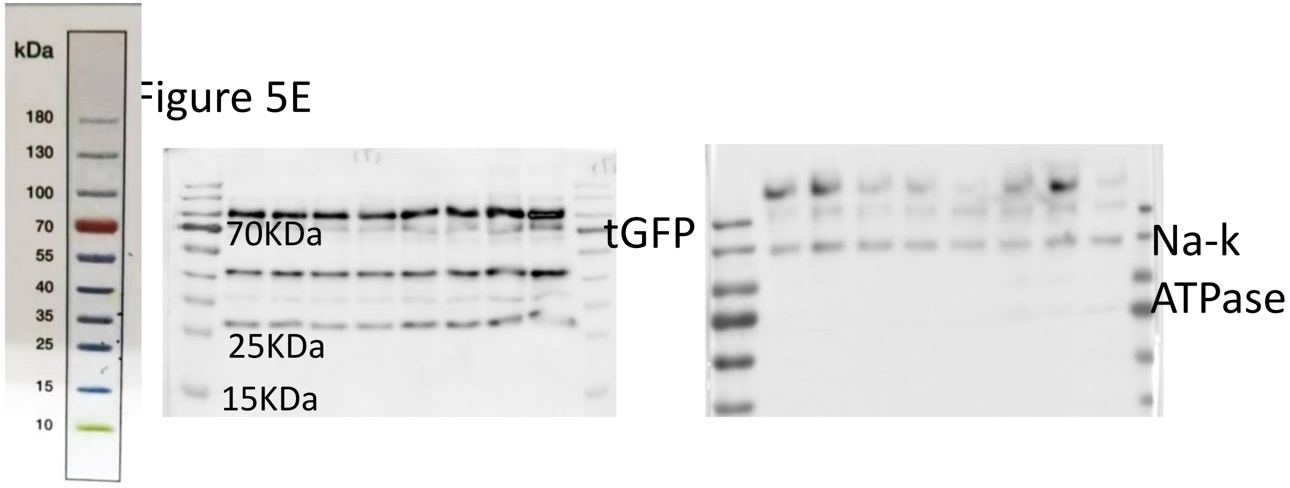

Figure 5G

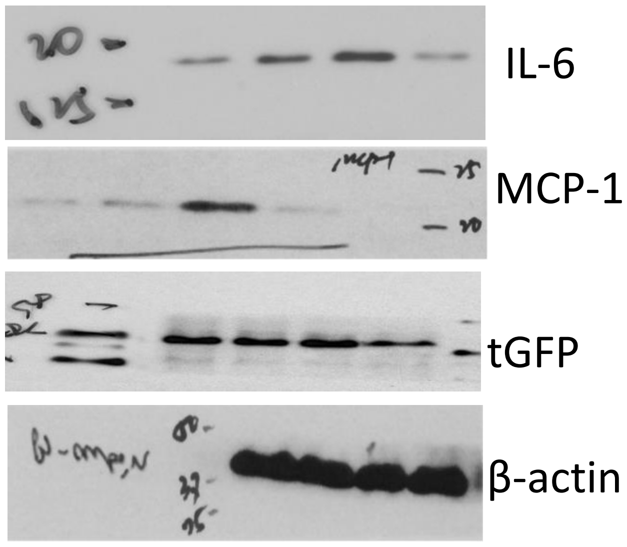

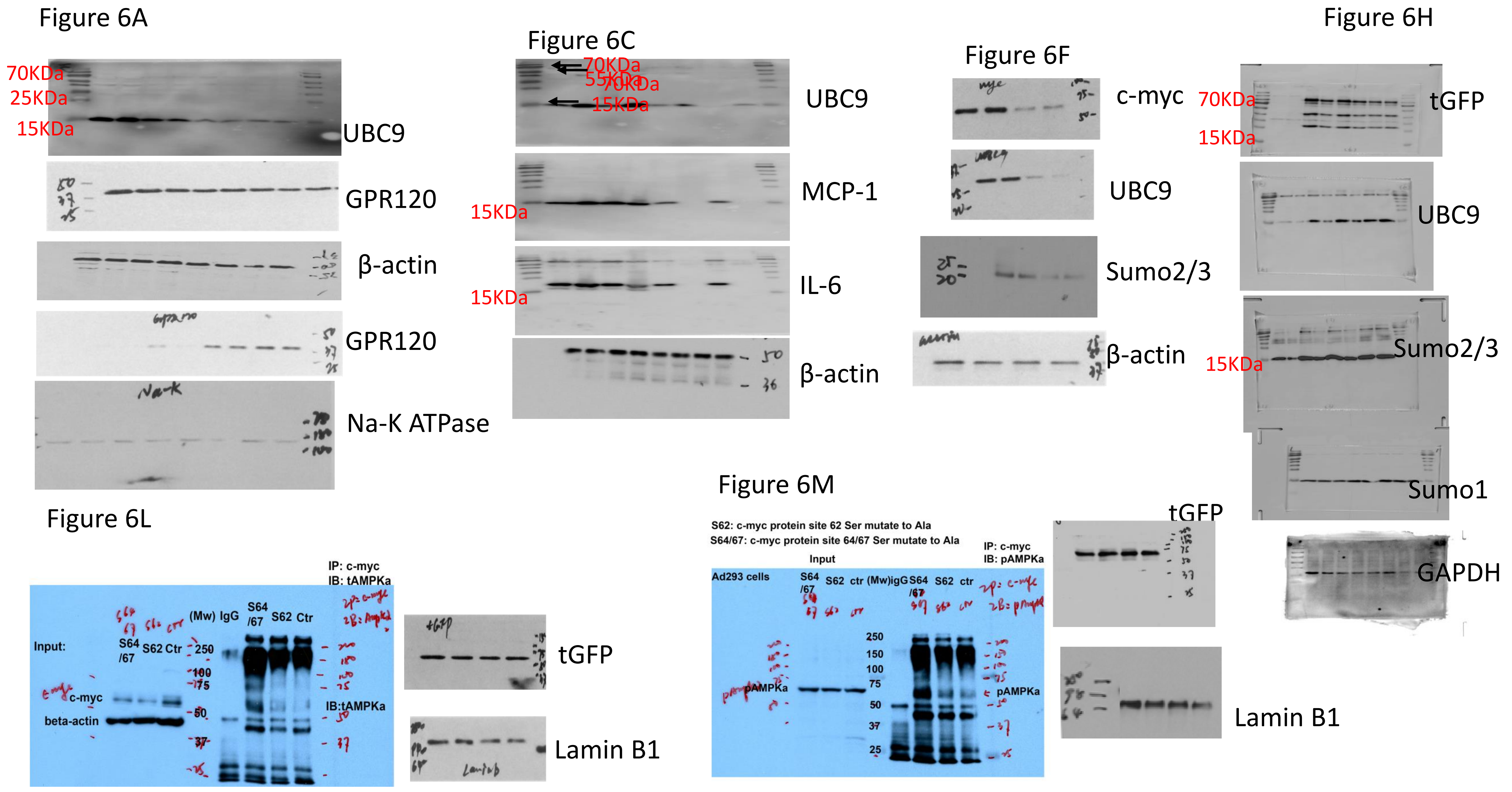

Figure 6A
70KDa
25KDa
15KDa
UBC9
GPR120
β-actin
GPR120
Na-K ATPase
Figure 6C
70KDa
55KDa
70KDa
15KDa
UBC9
15KDa
MCP-1
15KDa
IL-6
β-actin
Figure 6F
c-myc
UBC9
Sumo2/3
β-actin
Figure 6H
70KDa
15KDa
tGFP
UBC9
15KDa
Sumo2/3
Sumo1
GAPDH
Figure 6L
IP: c-myc
IB: tAMPKa
Input:
S64 /67
S62
Ctr
(Mw)
IgG
S64 /67
S62
Ctr
c-myc
beta-actin
250
100
75
50
37
IB:tAMPKa
tGFP
Lamin B1
Figure 6M
S62: c-myc protein site 62 Ser mutate to Ala
S64/67: c-myc protein site 64/67 Ser mutate to Ala
Input
IP: c-myc
IB: pAMPKa
Ad293 cells
S64 /67
S62
ctr
(Mw)
igG
S64 /67
S62
ctr
250
150
100
75
50
37
25
pAMPKa
pAMPKa
tGFP
Lamin B1

Figure 6O

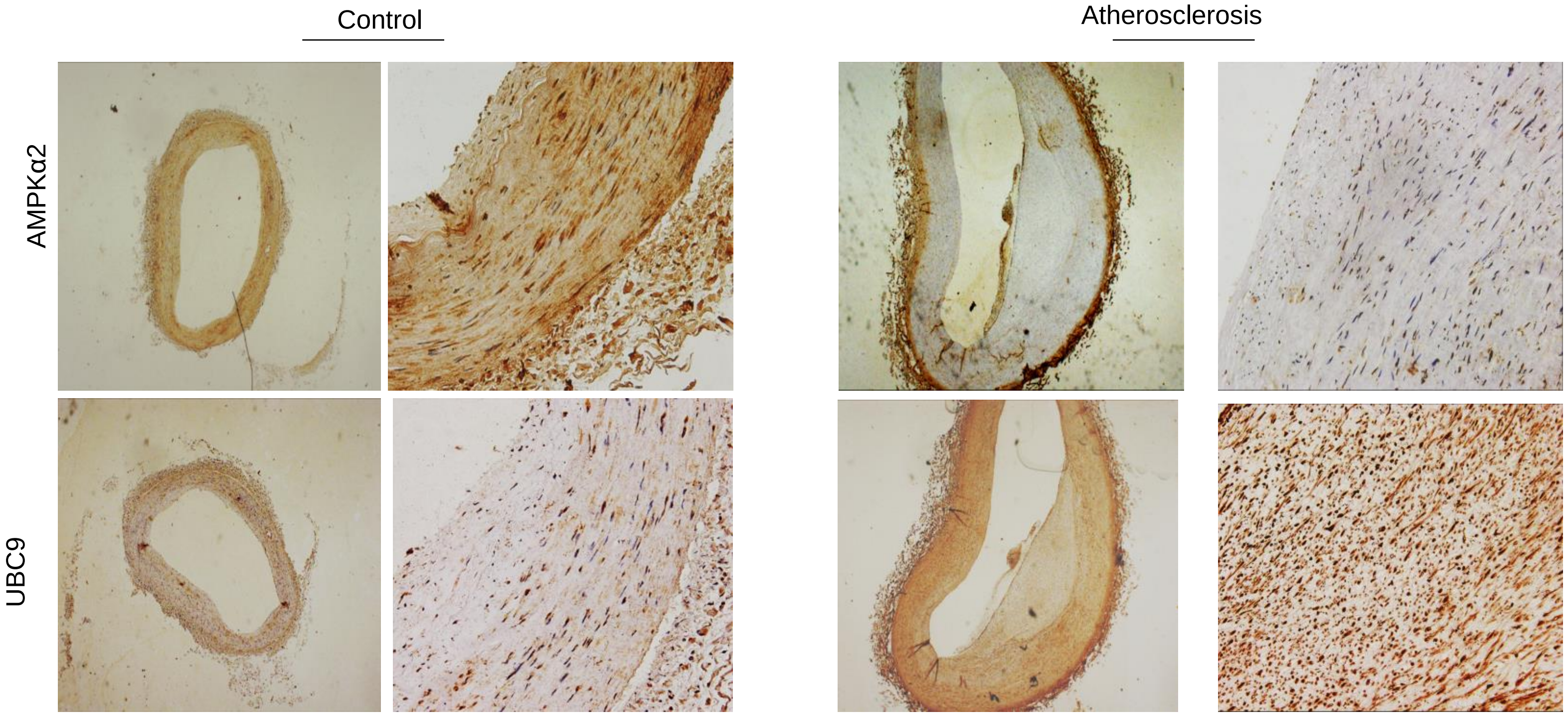

Supplemental Figure

S3A

A

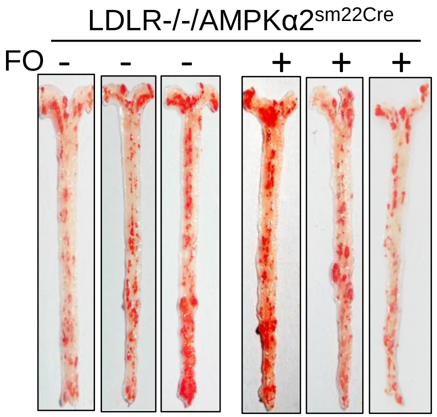

Supplemental Figure

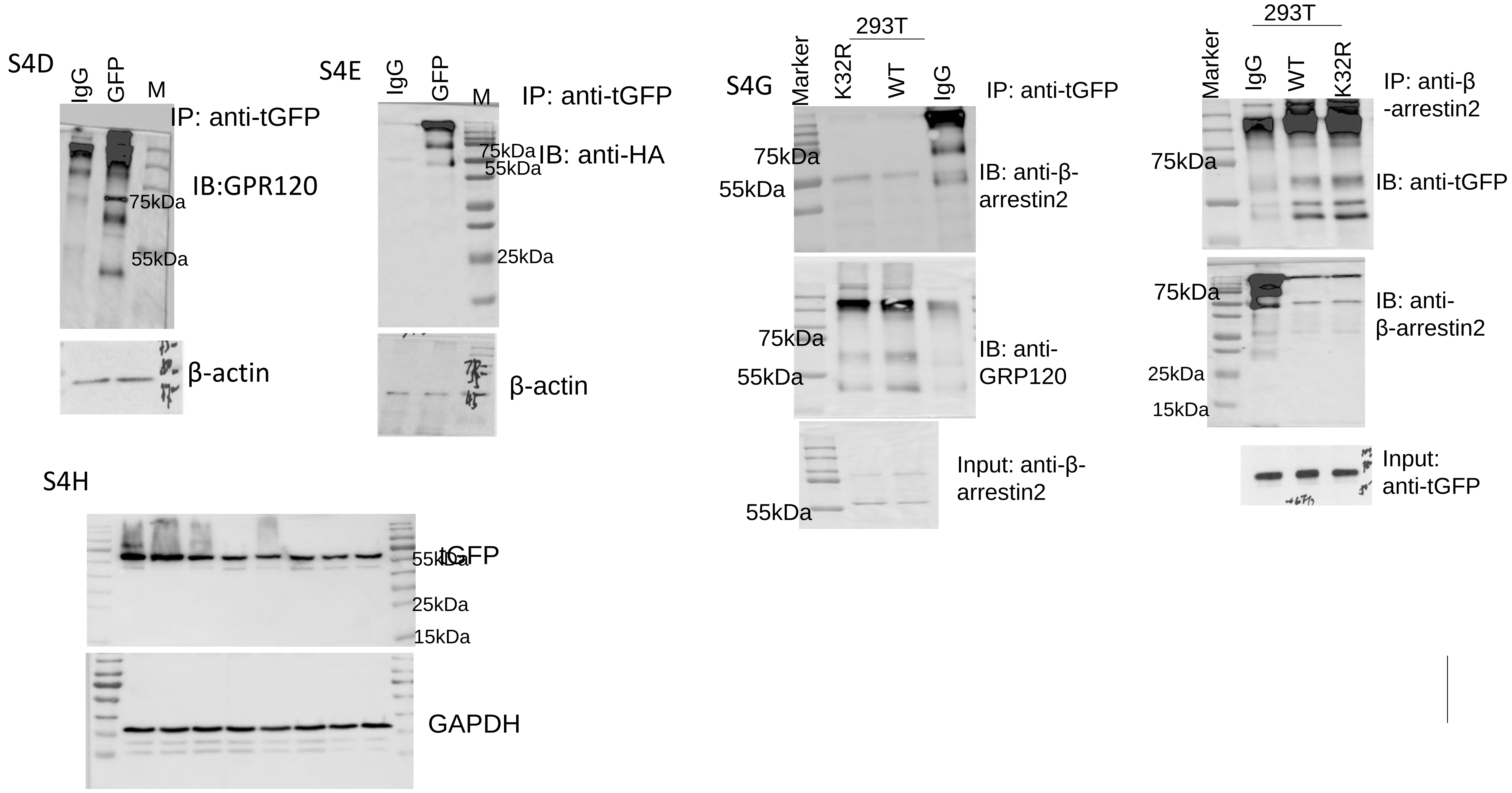

S5D

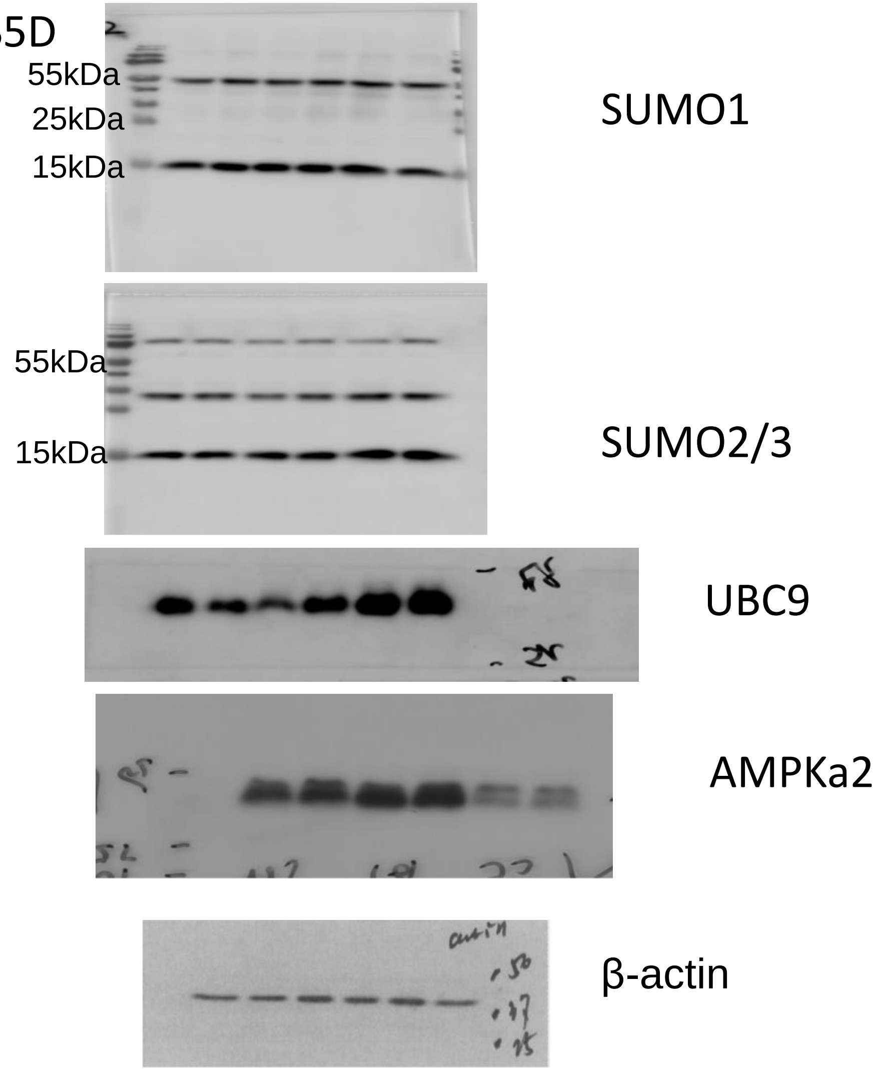
55kDa
25kDa
15kDa
SUMO1
55kDa
15kDa
SUMO2/3
UBC9
AMPKa2
β-actin

S5F

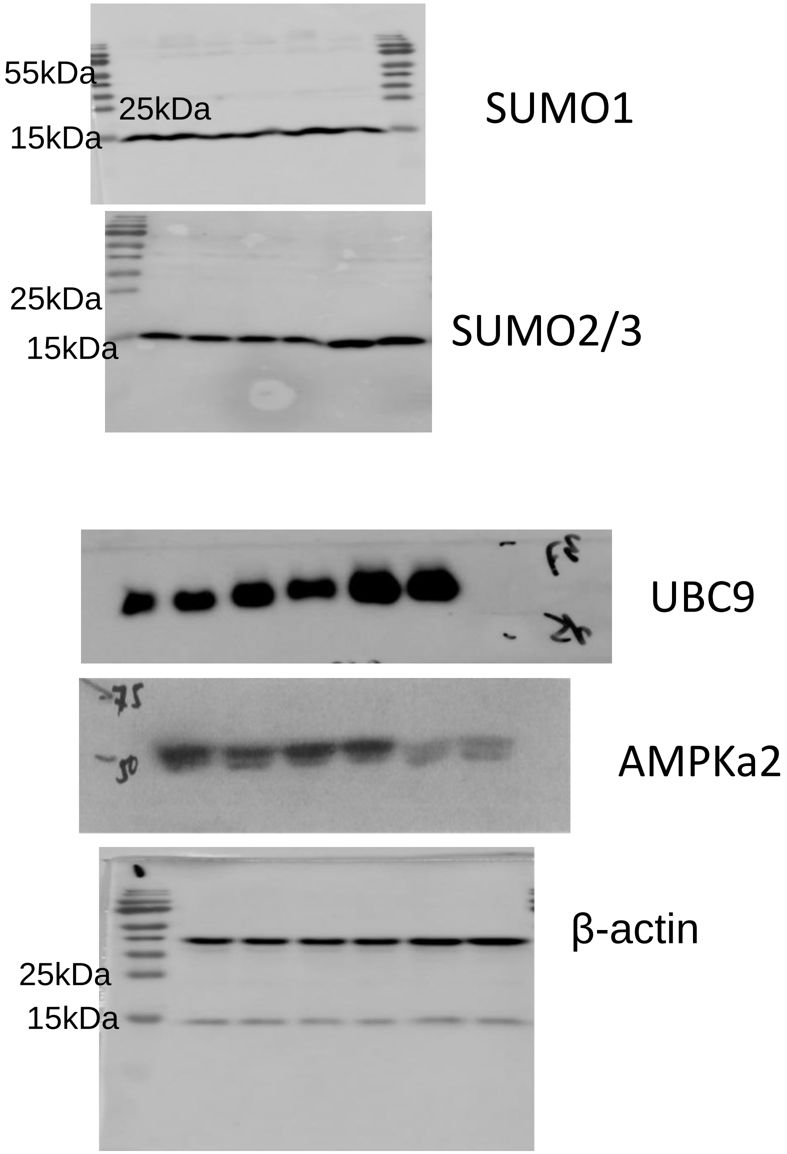
55kDa
25kDa
15kDa
SUMO1
25kDa
15kDa
SUMO2/3
UBC9
AMPKa2
25kDa
15kDa
β-actin

S6A

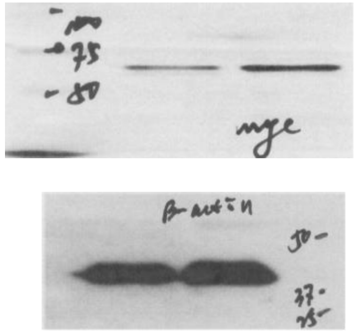

C-myc

β-actin

S6C

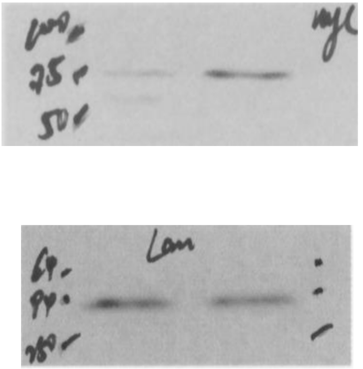

C-myc

Lamin B

S6E

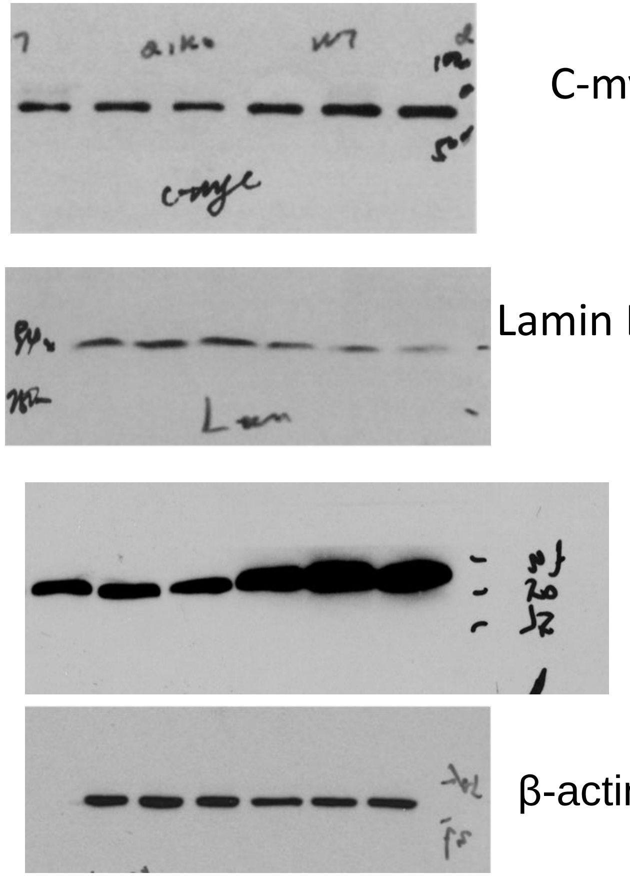

C-myc

Lamin B

C-myc

β-actin

S7C

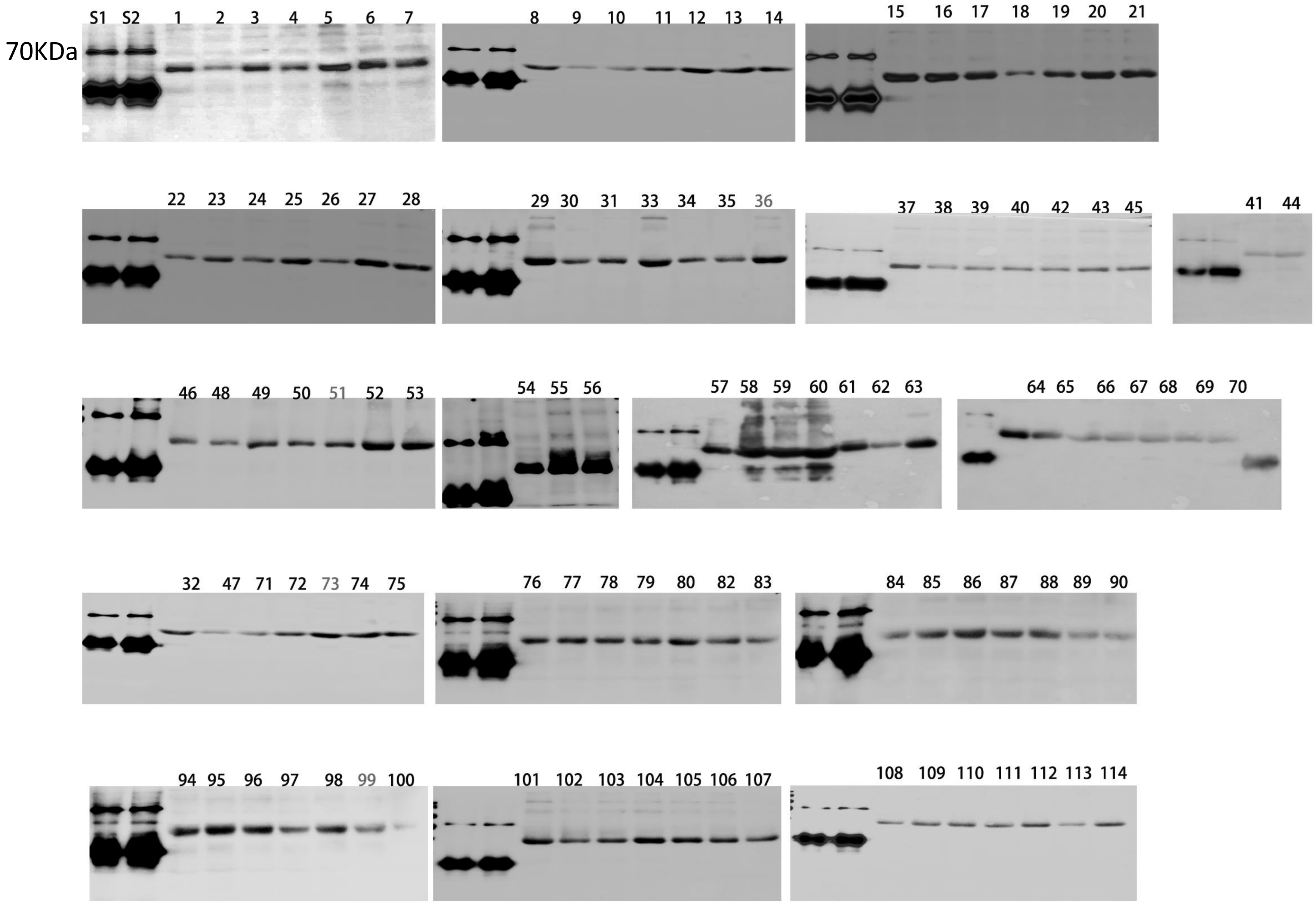
70KDa
S1 S2 1 2 3 4 5 6 7
8 9 10 11 12 13 14
15 16 17 18 19 20 21
22 23 24 25 26 27 28
29 30 31 33 34 35 36
37 38 39 40 42 43 45
41 44
46 48 49 50 51 52 53
54 55 56
57 58 59 60 61 62 63
64 65 66 67 68 69 70
32 47 71 72 73 74 75
76 77 78 79 80 82 83
84 85 86 87 88 89 90
94 95 96 97 98 99 100
101 102 103 104 105 106 107
108 109 110 111 112 113 114

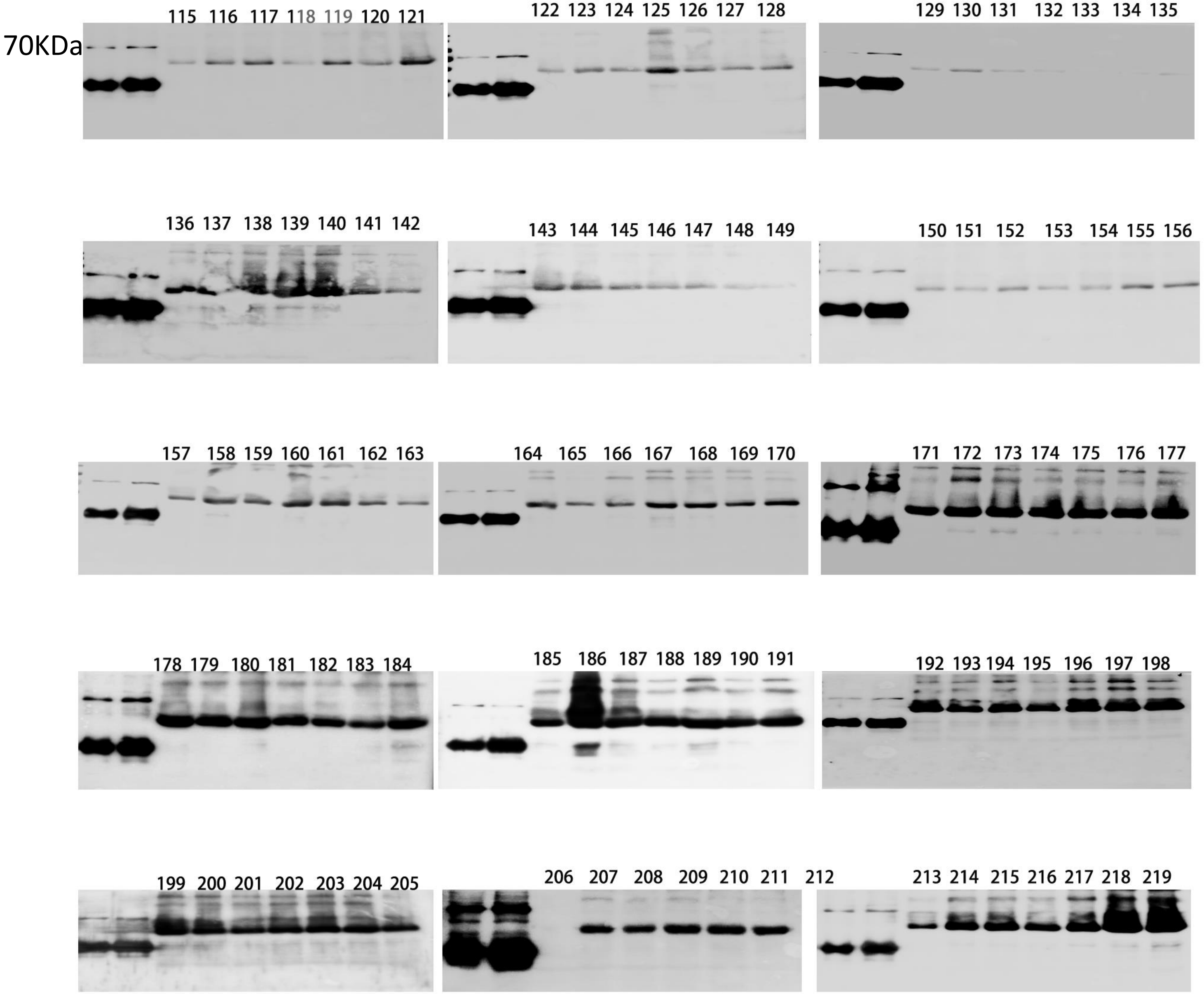
70KDa
115 116 117 118 119 120 121
122 123 124 125 126 127 128
129 130 131 132 133 134 135
136 137 138 139 140 141 142
143 144 145 146 147 148 149
150 151 152 153 154 155 156
157 158 159 160 161 162 163
164 165 166 167 168 169 170
171 172 173 174 175 176 177
178 179 180 181 182 183 184
185 186 187 188 189 190 191
192 193 194 195 196 197 198
199 200 201 202 203 204 205
206 207 208 209 210 211 212
213 214 215 216 217 218 219
